# Supplementary material for: Associations between lean maturity in primary care and musculoskeletal complaints among staff: a longitudinal study
Source: BMJ Open. 2023 Feb 22;13(2):e067753. doi: 10.1136/bmjopen-2022-067753 (PMC9950927; doi:10.1136/bmjopen-2022-067753)
Supplement: Author's manuscript [file bmjopen-2022-067753.draft_revisions.pdf]

BMJ Open is committed to open peer review. As part of this commitment we make the peer review history of every article we publish publicly available.

When an article is published we post the peer reviewers' comments and the authors' responses online. We also post the versions of the paper that were used during peer review. These are the versions that the peer review comments apply to.

The versions of the paper that follow are the versions that were submitted during the peer review process. They are not the versions of record or the final published versions. They should not be cited or distributed as the published version of this manuscript.

BMJ Open is an open access journal and the full, final, typeset and author-corrected version of record of the manuscript is available on our site with no access controls, subscription charges or pay-per-view fees (http://bmjopen.bmj.com).

If you have any questions on BMJ Open's open peer review process please email info.bmjopen@bmj.com

BMJ Open

Associations between Lean Maturity in primary care and musculoskeletal complaints among staff. A longitudinal study

Journal:	*BMJ Open*
Manuscript ID	bmjopen-2022-067753
Article Type:	Original research
Date Submitted by the Author:	26-Aug-2022
Complete List of Authors:	Kaltenbrunner, Monica; University of Gävle Faculty of Health and Occupational Studies, Department of Health and Caring Sciences<br>Mathiassen, Svend ; University of Gavle,<br>Bengtsson, Lars; University of Gävle, Department of Industrial Management<br>Högberg, Hans; University of Gävle, Department of Public Health and Caring Sciences<br>Engström, Maria; University of Gävle Faculty of Health and Occupational Studies, Faculty of Health and Occupational Studies; Uppsala University, Department of Public Health and Caring Sciences
Keywords:	PRIMARY CARE, Musculoskeletal disorders < ORTHOPAEDIC & TRAUMA SURGERY, Health & safety < HEALTH SERVICES ADMINISTRATION & MANAGEMENT

SCHOLARONE™
Manuscripts

I, the Submitting Author has the right to grant and does grant on behalf of all authors of the Work (as defined in the below author licence), an exclusive licence and/or a non-exclusive licence for contributions from authors who are: i) UK Crown employees; ii) where BMJ has agreed a CC-BY licence shall apply, and/or iii) in accordance with the terms applicable for US Federal Government officers or employees acting as part of their official duties; on a worldwide, perpetual, irrevocable, royalty-free basis to BMJ Publishing Group Ltd ("BMJ") its licensees and where the relevant Journal is co-owned by BMJ to the co-owners of the Journal, to publish the Work in this journal and any other BMJ products and to exploit all rights, as set out in our licence.

The Submitting Author accepts and understands that any supply made under these terms is made by BMJ to the Submitting Author unless you are acting as an employee on behalf of your employer or a postgraduate student of an affiliated institution which is paying any applicable article publishing charge ("APC") for Open Access articles. Where the Submitting Author wishes to make the Work available on an Open Access basis (and intends to pay the relevant APC), the terms of reuse of such Open Access shall be governed by a Creative Commons licence – details of these licences and which Creative Commons licence will apply to this Work are set out in our licence referred to above.

Other than as permitted in any relevant BMJ Author's Self Archiving Policies, I confirm this Work has not been accepted for publication elsewhere, is not being considered for publication elsewhere and does not duplicate material already published. I confirm all authors consent to publication of this Work and authorise the granting of this licence.

For peer review only

Associations between Lean Maturity in primary care and musculoskeletal complaints among staff. A longitudinal study

Monica Kaltenbrunner[1]RN, PhD, Svend Erik Mathiassen[1] PhD, Professor, Lars Bengtsson[2] PhD, Professor, Hans Högberg[3] Statistician, PhD, Maria Engström[3] RN, PhD, Professor.

[1]Centre for Musculoskeletal Research, Department of Occupational Health Sciences and Psychology, Faculty of Health and Occupational Studies, University of Gävle, Gävle, Sweden.

[2]Department of Industrial Management, Industrial Design and Mechanical Engineering, Faculty of Engineering and Sustainable Development, University of Gävle, Gävle, Sweden.

[3]Department of Caring Science, Faculty of Health and Occupational Studies, University of Gävle, Gävle, Sweden.

Correspondence to

Monica Kaltenbrunner; monica.kaltenbrunner.nykvist@hig.se

For peer review only

ABSTRACT

Objective We had two aims: 1) to determine the prevalence of musculoskeletal complaints among staff in primary care 2) to determine to what extent Lean maturity of the primary care unit can predict musculoskeletal complaints one year later.

Design Descriptive, correlational and longitudinal design.

Setting Primary care units in mid-Sweden.

Participants In 2015, staff members responded to a web survey addressing Lean maturity and musculoskeletal complaints. The survey was completed by 481 staff members (response rate 46%) at 48 units; 260 staff members at 46 units also completed the survey in 2016.

Outcome measures Associations with musculoskeletal complaints were determined both for Lean maturity in total, and for four Lean domains entered separately in a multivariate model, i.e., Philosophy, Processes, People and partners, and Problem-solving.

Results The shoulders (12-month prevalence 58%), neck (54%) and low back (50%) were the most common sites of 12-months retrospective musculoskeletal complaints at baseline. Shoulders, neck, and low back also showed the most complaints for the preceding 7 days (37%, 33% and 25%, respectively). The prevalence of complaints was similar at the 1-year follow-up. Total lean maturity in 2015 was not associated with musculoskeletal complaints, neither cross-sectionally nor one year later, for shoulders (one-year β: -0.002, 95% CI -.03 to .02), neck (β: 0.006, 95% CI -.01 to .03), low back (β: 0.004, 95% CI -.02 to .03) and upper back (β: 0.002, 95% CI -.02; .02).

Conclusion The prevalence of musculoskeletal complaints among primary care staff was high and did not change within a year. The extent of Lean maturity at the care unit was not associated with complaints among staff, neither in cross-sectional analyses nor in a one-year predictive analysis.

For peer review only

Keywords: Liker, Lean in Health care Questionnaire (LiHcQ), musculoskeletal disorders (MSD), Nordic Musculoskeletal Questionnaire (NMQ), pain

Strengths and limitations of this study

- The longitudinal design allowed analysis over time of associations between Lean maturity and self-reported musculoskeletal complaints.
- The convenience sample and the low response rate are potential limitations.
- Using self-reports may be a limitation, as responses may be biased by a positive or negative attitude toward Lean and the workplace.

INTRODUCTION

Implementation of Lean has become common in healthcare(1), with an aim to increase efficiency(2) and quality of care(3,4). Lean originates from the automotive industry(5) and has been described and construed in different ways, both there and in healthcare(1,4,6,7).

Several systems for describing Lean have been suggested. Liker's interpretation, which we use in the present study(5), includes 14 basic principles, which can be organized in four categories, in a so-called 4P model (Philosophy, Processes, People and partners, and Problem-solving). 'Philosophy' involves having a long-term perspective, focusing on the customer and having common goals that staff members strive after in order to create value for the customer, the organization and society as a whole. 'Processes' involve using resources optimally to improve flow in production. A good flow, obtained by, for instance, mapping processes, standardizing work and reducing waste, can increase quality and efficiency. 'People and partners' include showing respect and

For peer review only

enabling growth for individuals in the organization, as well as for suppliers and partners. ‘Problem-solving’ focusses on finding and solving the basic causes of problems that restrain flow and quality in production(5).

In Lean, staff are given responsibility, and they are expected to contribute to decision-making, and to engage in securing continuous improvements. Customer satisfaction is paramount, and according to Liker, all staff throughout the organization need to be aware of that and contribute to achieving it(5). Liker also claims that all Lean principles must be adopted throughout the organization in order to fully reach the desired results(5). Thus, the ultimate level of Lean maturity will be obtained when all staff adopt all Lean principles to their full extent(8).

In Sweden, Lean was first implemented in industry, and began to influence healthcare management in the mid-90s(9). A number of studies, a majority of which in hospitals both in Sweden and internationally, suggest that Lean has been adopted only in part, with emphases on, e.g. standardized work, enhanced seamlessness and flow, and problem-solving (1,4,5,10,11). Some positive results of adopting Lean have been reported, such as Lean improving working conditions(12) and patient safety(13,14). In a previous study in primary care, we showed that Lean maturity had a positive association with quality of care as perceived by staff(8). However, other studies conclude that effects of implementing Lean are ambiguous(15,16). In the context of work-related complaints, Lean needs, according to Liker(5), to address human resources in the sense that Lean should not contribute to excessive loads on the staff, with might lead to negative consequences to health, including, e.g. musculoskeletal complaints(17). However, two reviews(17,18) and one overview(19) suggest that Lean may, indeed, contribute to

For peer review only

increased musculoskeletal complaints, at least in other sectors than healthcare. Lean may influence working conditions so that work demands and work pace increase and, thus, opportunities to recover decrease, which in turn may increase the risk for musculoskeletal complaints(17).

Little is known, however, about associations between Lean and musculoskeletal complaints specifically in healthcare(1,2,10). Musculoskeletal complaints among healthcare staff have been reported to be common (22), and a concern(23). For instance, a review of musculoskeletal complaints among registered nurses, midwifes and physicians working predominantly in hospitals, reported that 45% had musculoskeletal complaints in the neck, 40% in the shoulders and 35% in the upper back during a 12-month period, as assessed using the Nordic Musculoskeletal Questionnaire (NMQ)(21). In a cross-sectional study by Freimann et al.(24) among Finnish nurses, also based on the NMQ, the 12-month prevalence of musculoskeletal complaints was 57% for the low back, 56% for the neck and 31% for the shoulders.

In healthcare at hospitals and in eldercare, likely risk factors for musculoskeletal complaints are awkward postures, repetitive work, and heavy lifting(20,22,24,25,26–29), the effects of which may be modified by female gender, older age, and larger BMI(30). Primary care, however, represents working conditions differing from those in hospitals. In Sweden, primary care offers prevention, advice and treatment to the citizens and it is the first level of care in the healthcare system(25). Work in primary care has been described to have low physical demands, while cognitive demands are substantial(31). Psychosocial working conditions such as low decision authority, low social support high job demands, high job strain and psychological distress have been described as risk

For peer review only

factors for MSDs(32) and these conditions may prevail in primary care. Thus, the panorama of risk factors for musculoskeletal complaints, as well as their prevalence, may well differ between primary care and other healthcare sectors, leading to a different prevalence of complaints.

Few studies have, however, investigated this issue. A cross-sectional study in Portuguese primary care, based on the NMQ, reported that 63% of the participating nurses had low back pain, 50% neck pain, and 41% pain in the dorsal region during the preceding 12-month period(26). In Swedish primary care, the prevalence of musculoskeletal complaints has not, to our knowledge, been assessed in any scientific study. Since Lean sets the scene for working conditions among staff, the association between Lean maturity and musculoskeletal complaints in primary care stands up as an interesting issue for research. Hence, the aim of the present study was twofold: 1) to determine the prevalence of musculoskeletal complaints among staff in primary care and 2) to determine to what extent Lean maturity of the primary care unit could predict musculoskeletal complaints one year later.

METHOD

Primary care units

The study had a descriptive, correlational and longitudinal design. A convenience sample of 52 primary care units located in a region in mid-Sweden were first approached; 42 units accepted, of which 38 were public primary care units and four belonged to private providers. Due to this shortage of private care units, one of the largest nationwide private health care providers (organizing 85 primary care units) was approached; six units from there agreed to participate, in addition to the four already recruited. Units were required

For peer review only

to, according to their own opinion, have adopted Lean to some extent to be eligible for participation in the study.

Participants

All staff at the recruited units were asked to participate, and responded to a questionnaire in May 2015 (T1) and again in May 2016 (T2), with an inclusion criterion of having worked at the unit at least three months prior to data collection. The number of staff eligible at T1 was 1040; 481 responded, distributed among 48 units, i.e. a 46% response rate. At T2, 406 staff members of those who responded at T1 were still eligible. In total, 260 staff members at 46 units responded at both T1 and T2 (Figure 1).

-Insert Figure 1 about here-

Data collection procedure

Data were collected at T1 and T2 using a web survey. After two non-successful reminders, non-responders were sent a paper version. Confidentiality and voluntary participation were emphasized at data collection. Consent to participate was given when participants completed and returned the questionnaire. The study is part of a larger research project on Lean maturity in primary care; data have been used and published for other purposes in previous papers(8,33,34).

Measurements

Staff members' self-rated perception of *Lean maturity* at their unit was assessed using the 16-item Lean in Health care Questionnaire (LiHcQ), shown to have acceptable psychometric properties(33). The LiHcQ is based on the 4P model of Lean proposed by

For peer review only

Liker(5), addressing Philosophy (3 items), Processes (7 items), People and partners (3 items), and Problem-solving (3 items). Answers are provided on a scale ranging from 1, indicating low Lean maturity, to 5, indicating high Lean maturity. Thus, the total LiHcQ score could range from 16 to 80. In the present study, Cronbach's alpha was 0.93 for total Lean maturity, and for Philosophy 0.75, Processes 0.86, People and partners 0.60, and Problem-solving 0.81, respectively. *Musculoskeletal complaints* (pain and/or discomfort) were assessed using the Nordic Musculoskeletal Questionnaire, NMQ(20). All 18 items in the first part of the NMQ were included; they address, with response alternatives "yes" and "no", the occurrence of musculoskeletal complaints during the past 12-month and 7-day periods in nine body regions (neck, shoulders, low back, upper back, elbows, hands/wrists, hips, knees and ankles/feet). In the second part of the NMQ (which in the present study addressed pain only in the neck, shoulders, and low back), the respondent is asked to respond with a "yes" or "no" to whether she, during the preceding year, has been limited in performing activities during work or leisure, has changed jobs or work tasks, or has visited a health practitioner due to musculoskeletal complaints. Items also address the total duration of musculoskeletal complaints during the preceding year (5-point scale, with a 1 for 0 days and a 5 for every day). Participants were also asked to report gender, age, height, weight, years worked in the profession, and years worked at the present primary care unit. Lean maturity as well as musculoskeletal complaints were assessed both at T1 and T2.

Statistical analysis

Data analyses were conducted using IBM SPSS Statistics, version 24. Descriptive statistics were used for demographic data, the NMQ and LiHcQ. Differences between T1 and T2 in musculoskeletal complaints were analyzed using McNemar's test; changes in

For peer review only

Lean maturity over time were analyzed using a paired *t*-test. Associations between variables in the cross-sectional as well as the longitudinal case were determined using Generalized estimating equations (GEE), with an exchangeable correlation structure accounting for the correlated nature of data collected at the same primary care unit. Predictive analyses over time were conducted using LiHcQ scores at T1 as the predictor variable, and musculoskeletal complaints at T2 as the outcome, controlling for complaints at T1. Additional control variables were gender, BMI, and age categorized in quartiles. Since musculoskeletal complaints showed to be most prevalent in shoulders, neck, low back and upper back, these body regions were selected for analysis of associations between Lean maturity and complaints. Two models were tested based on cross-sectional data, and four models based on longitudinal data (Table 1).

Table 1 Variables included in the six models addressing associations between Lean maturity and musculoskeletal complaints. All six models included gender, Body Mass Index and age (in quartiles) as control variables.

	**Cross-sectional data**		**Longitudinal data**			
	Model 1a	Model 1b	Model 2a	Model 2b	Model 2c	Model 2d
**Independent variables**						
Lean maturity at T1, in total	X		X	X		
Lean maturity at T1, each of the 4P		X			X	X
**Dependent variables**						
Complaints at T1	X	X		X		X
Complaints at T2			X	X	X	X

T1, time 1 (2015); T2, time 2 (2016);
4P: Philosophy, Processes, People and partners, and Problem-solving;

Missing data were handled using multiple imputation (MI; 35). When conducting a MI, five new datasets are produced with imputed data. In the subsequent analysis, results from the original dataset and the five MI datasets are generated, as well as a pooled result. We report the pooled results, in terms of regression coefficients with 95%

confidence intervals (CI) and corresponding p-values. Standard deviations (SD) and odds ratios (OR) were obtained on basis of the five MI datasets, since pooled results do not provide these metrics.

Patient and public involvement

No patients were involved.

For peer review only

RESULTS

Participants

The sample at T1 consisted mostly of females, their mean age was 50.2 years (SD 10.3) (Table 2). The most common profession was registered nurse, and most of the participants worked in public primary care units. Non-responders at T2, i.e. those only responding at T1, did not differ significantly from those responding at both T1 and T2 with respect to gender, age, years worked in the profession and years worked at the present unit.

Table 2 Participant characteristics

	T1 N:481	(%)	T2 N:260	(%)
**Participants working at**				
Public primary care units	407	(85)	216	(83)
Private primary care units	74	(15)	44	(17)
**Gender**				
Women	422	(88)	224	(86)
Men	59	(12)	35	(13)
**Profession**				
Registered Nurses	181	(38)	101	(39)
Physician	70	(15)	37	(14)
Administrator and secretary	64	(13)	31	(12)
Physiotherapist	47	(10)	30	(12)
Licensed Practical Nurse	45	(9)	24	(9)
Social worker and psychologist	41	(9)	21	(8)
Manager	26	(5)	17	(7)
Occupational therapist	17	(4)	8	(3)
Dietician	3	(1)	1	(< 1)

	T1	T2
**Age**		
Mean (SD)	50.2 (10.3)	50.6 (10.0)
Md (Q_1-Q_3)	52.0 (44.0-59.0)	53.0 (44.0-59.0)
**BMI**		
Mean (SD)	25.0 (4.0)	25.4 (4.1)
Md (Q_1-Q_3)	24.2 (22.4-27.4)	24.6 (22.6-27.8)
**Years worked in the profession**		
Mean (SD)	21.5 (12.1)	22.0 (11.8)
Md (Q_1-Q_3)	21.0 (11.0-31.0)	20.0 (13.0-32.0)
**Years worked at the present unit**		
Mean (SD)	9.1 (9.0)	9.3 (8.7)
Md (Q_1-Q_3)	5.0 (2.0-14.0)	5.0 (3.0-14.0)

T1, time 1 (2015); T2, time 2 (2016); BMI, body mass index; Md, median. Numbers regarding profession do not add up to 481 and 260 because some participants had multiple professions.

Prevalence of musculoskeletal complaints

At T1, 50% to 58% of the participants (n=481) reported musculoskeletal complaints the preceding 12-month period in the shoulder, neck or low back (Table 3). Musculoskeletal complaints were also most common in these three body regions when reported for the preceding seven days (Table 4). The same pattern was found when analyzing only those who responded at both T1 and T2 (n=260).

Table 3 Prevalence of musculoskeletal complaints at T1 (n=481). Data concern the preceding 12-month period if not stated otherwise.

	Musculo-skeletal complaints	Musculo-skeletal complaints during the preceding 7-day period	Musculoskeletal complaints limiting activities during…* ..leisure	..work	Changed job or work tasks due to musculo-skeletal complaints *	Visited a health practitioner due to musculo-skeletal complaints *
	n (%)	n (%)	n (%)	n (%)	n (%)	n (%)
Shoulders	264 (58%)	176 (37%)	86 (18%)	30 (6%)	23 (5%)	86 (18%)
Neck	249 (54%)	157 (33%)	77 (16%)	35 (7%)	15 (3%)	75 (16%)
Low back	222 (50%)	119 (25%)	102 (21%)	44 (9%)	33 (7%)	70 (15%)
Upper back	160 (36%)	74 (15%)				
Wrists/hands	133 (30%)	81 (17%)				
One hip or both hips	121 (27%)	72 (15%)				
One knee or both knees	121 (27%)	71 (15%)				
One or both ankles/feet	112 (25%)	76 (16%)				
Elbows	54 (12%)	29 (6%)				

* These outcomes were obtained only for shoulders, neck and low back, as explained in the running text. All percentages refer to n=481.

For peer review only

At T1 (n=481), musculoskeletal complaints during the preceding 12-month period most often lasted for 1-7 days for the low back (21%) and neck (20%), while shoulder complaints most often lasted for more than 30 days (15%). Nine percent reported daily musculoskeletal complaints the past 12-month period in the shoulders, 7% in the low back, and 6% in the neck.

Changes in musculoskeletal complaints

Changes over time in musculoskeletal complaints were analyzed for shoulders, neck, low back, and upper back. Between 20% and 43% of the participants reported musculoskeletal complaints from these body regions at both T1 and T2 (Table 4). Between 12% and 13% reported musculoskeletal complaints in the four body regions at T1 which were not present anymore at T2. Between 11% and 14% had no musculoskeletal complaints at T1, but reported complaints at T2. Most of the participants (72% to 76%) did not change their ratings between T1 and T2. Differences between T1 and T2 in musculoskeletal complaints were non-significant for all four body regions (Table 4).

Table 4 Combined prevalence of musculoskeletal complaints at T1 and T2, including results of McNemar's test for differences in ratings between T1 and T2

	**Shoulders** (n=260)	**Neck** (n=260)	**Low back** (n=260)	**Upper back** (n=260)
Musculoskeletal complaints at T1 and T2	110 (42%)	113 (43%)	94 (36%)	51 (20%)
Musculoskeletal complaints at T1 but not at T2	33 (13%)	31 (12%)	34 (13%)	32 (12%)
No musculoskeletal complaints at T1 but complaints at T2	37 (14%)	32 (12%)	37 (14%)	29 (11%)
Musculoskeletal complaints neither at T1 nor at T2	80 (31%)	84 (32%)	94 (36%)	147 (57%)
McNemar test, original dataset, P	0.892	0.609	0.775	0.788
MI-dataset 1-5, P range	0.899 to 1.000	0.470 to 0.906	0.366 to 1.000	0.550 to 1.000

T1, time 1; T2, time 2. MI, multiple imputation. P calculated with binomial distribution

For peer review only

Lean maturity

Mean Lean maturity was 46.2 at T1 (SD ranging from 11.5 to 11.7 in the MI dataset), and 45.3 at T2 (SD ranging from 11.9 to 12.1). Between T1 and T2, 94.5% of all participants changed their rating of total Lean maturity, however these changes were both positive and negative (Figure 2). Thus, no statistically significant difference in Lean maturity was found at the group level between T1 and T2 (pooled data; p=.16).

-Insert Figure 2 about here-

Associations between Lean maturity and musculoskeletal complaints

Cross-sectional data at T1 showed no statistically significant associations between Lean maturity in total and musculoskeletal complaints in the shoulders, neck, low back or upper back (p between .09 and .96; Table 5, Model 1a). Using each of the 4P as the independent variable instead of Lean maturity in total did not result in any statistically significant associations either, except for neck complaints showing a positive association with Philosophy (p= <.01; Table 5, Model 1b) and a negative association with People and partners (p=.03).

Table 5 Cross-sectional associations between Lean maturity and musculoskeletal complaints in the shoulders, neck, low back and upper back.

	Model 1a				Model 1b			
	β	95% CI	*P*	OR range; MI data set 1-5	β	95% CI	*P*	OR range; MI data set 1-5
**Shoulder complaints**<br>Intercept -0.61					Intercept -0.80			
**Lean maturity in total**	-0.02	-0.04; 0.01	.17	0.98; 0.99				
**Philosophy**					0.12	-0.02; 0.26	.09	1.09; 1.17
**Processes**					0.01	-0.13; 0.15	.89	0.97; 1.09
**People and partners**					-0.16	-0.34; 0.03	.10	0.83; 0.89

For peer review only

**Problem-solving**					-0.08	-0.33; 0.17	.49	0.81; 0.98
**Control variables**								
**Female gender**	0.91	0.13; 1.69	**.02**	2.32; 2.62	0.88	0.07; 1.69	**.03**	2.20; 2.59
**BMI**	0.03	-0.05; 0.10	.51	1.02; 1.04	0.03	-0.06; 0.11	.56	1.02; 1.03
**Age 43 to 51 years**	0.20	-0.56; 0.97	.61	1.15; 1.37	0.23	-0.58; 1.04	.58	1.13; 1.43
**52 to 57 years**	0.74	-0.20; 1.67	.12	2.02; 2.14	0.85	-0.16; 1.86	.10	2.13; 2.80
**58 years and older**	-0.22	-0.10; 0.55	.58	0.77; 0.86	-0.22	-1.04; 0.59	.59	0.76; 0.86
**Neck complaints** Intercept -0.34					Intercept -.60			
**Lean maturity in total**	-0.01	-0.03; 0.01	.50	0.99; 1.00				
**Philosophy**					0.19	0.07; 0.31	**<.01**	1.19; 1.23
**Processes**					-0.02	-0.13; 0.08	.66	0.95; 1.00
**People and partners**					-0.19	-0.36; -0.02	.03	0.78; 0.85
**Problem-solving**					-0.00	-0.22; 0.21	.97	0.93; 1.06
**Control variables**								
**Female gender**	1.02	-0.05; 2.09	.06	2.60; 3.05	1.06	-0.07; 2.18	.07	2.51; 3.36
**BMI**	0.02	-0.04; 0.08	.55	1.01; 1.03	0.03	-0.04; 0.09	.41	1.02; 1.04
**Age 43 to 51 years**	-0.07	-0.81; 0.67	.85	0.92; 0.95	-0.12	-0.88; 0.64	.76	0.84; 0.92
**52 to 57 years**	-0.90	-1.65; -0.15	**.02**	0.39; 0.43	-0.94	-1.74; -0.13	**.02**	0.36; 0.46
**58 years and older**	-0.97	-1.77; -0.17	**.02**	0.36; 0.40	-1.05	-1.93; -0.18	**.02**	0.32; 0.37
**Low back complaints** Intercept -1.44					Intercept -1.59			
**Lean maturity in total**	-0.02	-0.04; 0.00	.09	0.98; 0.98				
**Philosophy**					0.08	-0.06; 0.21	.29	1.06; 1.12
**Processes**					0.00	-0.11; 0.12	.97	0.95; 1.04
**People and partners**					-0.14	-0.33; 0.05	.14	0.83; 0.92
**Problem-solving**					-0.05	-0.29; 0.19	.66	0.89; 1.07
**Control variables**								
**Female gender**	0.28	-0.56; 1.12	.51	1.18; 1.66	0.26	-0.65; 1.17	.57	1.08 1.66
**BMI**	0.08	0.01; 0.15	**.02**	1.08; 1.09	0.08	0.01; 0.16	**.03**	1.07; 1.10
**Age 43 to 51 years**	0.39	-0.43; 1.20	.35	1.34; 1.59	0.40	-0.40; 1.20	.33	1.38; 1.55
**52 to 57 years**	-0.28	-1.20; 0.65	.56	0.58; 0.88	-0.21	-1.24; 0.83	.69	0.56; 0.98
**58 years and older**	-0.22	-0.96; 0.52	.56	0.68; 0.91	-0.22	-0.99;	.58	0.67; 0.92

For peer review only

**Upper back complaints** Intercept -1.96					Intercept -2.01			
**Lean maturity in total**	0.00	-0.02; 0.02	.96	1.00; 1.00				
**Philosophy**					.01	-0.11;	.82	0.96; 1.05
**Processes**					.02	-0.09; 0.12	.77	0.98; 1.03
**People and partners**					.04	-0.14; 0.23	.65	1.01; 1.10
**Control variables**								
**Female gender**	0.75	-0.21; 1.70	.13	1.88; 2.34	0.69	-0.29; 1.67	.17	1.78; 2.27
**BMI**	0.03	-0.04; 0.10	.40	1.02; 1.03	0.03	-0.04; 0.10	.44	1.02; 1.04
**Age 43 to 51 years**	-0.25	-0.89; 0.40	.46	0.74; 0.85	-0.22	-0.89; 0.45	.52	0.76; 0.86
**52 to 57 years**	-0.10	-0.99; 0.79	.83	0.83; 1.01	-0.05	-0.98; 0.88	.92	0.87; 1.06
**58 years and older**	-0.43	-1.21; 0.36	.28	0.60; 0.71	-0.40	-1.21; 0.41	.33	0.60; 0.74

Reference in the control variables were male and age ≤ 42. OR, odds ratio; 4P, Philosophy, Processes, People and partners, and Problem-solving; β, unstandardized beta coefficient; CI, confidence interval; MI, multiple imputation; P, p-value; BMI, body mass index

Similarly, when analyzing longitudinal data, we found no statistically significant associations between Lean maturity in total at T1 and musculoskeletal complaints in the four body regions at T2 (p from .57 to .88) (Table 6, 7, 8 and 9). When analyzing associations between complaints at T2 and each of the individual 4P at T1, adding complaints at T1 as a covariate, People and partners were positively associated with neck complaints (p=.03) (Table 7, model 2d).

For peer review only

Table 6 Association between Lean maturity and musculoskeletal complaints in the shoulders over time. Predictors were Lean maturity in total at T1 (models 2a, 2b), or each of the 4Ps (models 2c, 2d); complaints at T2 were the outcome in all models. Gender, BMI and age were included as covariates in all models.

**Shoulder complaints at T2**	**Model 2a, only Lean maturity in total and covariates**				**Model 2b, complaints T1 added**				**Model 2c, each of the 4P**				**Model 2d, complaints T1 added**			
	β	95% CI	*P*	OR range; MI data set 1-5	β	95% CI	*P*	OR range; MI data set 1-5	β	95% CI	*P*	OR range; MI data set 1-5	β	95% CI	*P*	OR range; MI data set 1-5
Intercept				-0.29				-1.02				-0.43				-1.12
Lean maturity in total at T1	-0.002	-0.03; 0.02	.85	0.996; 1.00	0.006	-0.02; 0.03	.61	1.00; 1.10								
Shoulder complaints at T1					2.00	1.36; 2.66	**<.01**	6.83; 8.07								
Philosophy									0.07	-0.05; 0.19	.23	1.06; 1.09	0.03	-0.11; 0.16	.70	1.05; 1.01
Processes									-0.03	-0.13; 0.08	.64	0.95; 1.01	-0.04	-0.14; 0.07	.51	0.95; 1.00
People and partners									-0.01	-0.18; 0.15	.88	0.96; 1.01	0.07	-0.11; 0.25	.46	1.04; 1.10
Problem-solving									-0.02	-0.19; 0.16	.85	0.92; 1.03	0.02	-0.13; 0.17	.80	0.98; 1.06
Complaints at T1													2.02	1.36; 2.68	**<.01**	6.72; 8.22
Female gender	0.29	-0.26; 0.83	.30	1.31; 1.36	-0.14	-0.69; 0.41	.61	0.83; 0.93	0.27	-0.28; 0.82	.34	1.22; 1.36	-0.14	-0.72; 0.43	.62	0.81; 0.96
BMI	0.02	-0.05; 0.08	.63	1.00; 1.03	0.01	-0.06; 0.07	.85	0.99; 1.02	0.02	-0.05; 0.09	.55	1.00; 1.04	0.01	-0.06; 0.08	.69	0.99; 1.03
Age 43 to 51 years	0.03	-0.74; 0.80	.94	0.96; 1.11	-0.08	-0.98; 0.82	.87	0.86; 1.01	0.03	-0.76; 0.82	.942	0.95; 1.09	-0.08	-1.00; 0.83	.86	0.87; 1.00
52 to 57 years	0.34	-0.52; 1.20	.44	1.29; 1.51	0.02	-0.92; 0.96	.96	0.92; 1.12	0.34	-0.56; 1.24	.456	1.24; 1.48	-0.03	-0.99; 0.94	.96	0.86; 1.05
58 years and older	-0.26	-0.94; 0.43	.47	0.73; 0.84	-0.20	-0.86; 0.46	.56	0.76; 0.92	-0.27	-0.98; 0.44	.454	0.71; 0.86	-0.21	-0.87; 0.45	.52	0.76; 0.90

Reference in the control variables were male and age ≤ 42; β, unstandardized beta coefficient; T1, time 1; T2 time, 2; 4P, Philosophy, Processes, People and partners, and Problem-solving; CI, confidence interval; P, p-value; OR, odds ratio; MI, multiple imputation.

Table 7 Association between Lean maturity and musculoskeletal complaints in the neck over time. Predictors were Lean maturity in total at T1 (models 2a and 2b) or each of 4P's (model 2c and 2d); complaints at T2 were the outcome in all models. Gender, BMI and age were included as covariates in all models.

**Neck complaints at T2**	**Model 2a, only Lean maturity in total and covariates**				**Model 2b, complaints T1 added**				**Model 2c, 4P (instead of Lean maturity in total)**				**Model 2d, complaints T1 added**			
	β	95% CI	*P*	OR range; MI data set 1-5	β	95% CI	*P*	OR range; MI data set 1-5	β	95% CI	*P*	OR range; MI data set 1-5	β	95% CI	*P*	OR range; MI data set 1-5
Intercept				-0.36				-1.53				-0.60				-1.74
Lean maturity in total at T1	0.006	-0.01; 0.03	.57	1.00; 1.01	0.01	-0.01; 0.04	.28	1.01; 1.02								
Neck complaints at T1					2.31	1.66; 2.96	**<.01**	9.54; 10.71								
Philosophy									0.12	-0.02; 0.25	.08	1.09; 1.15	0.03	-0.10; 0.17	.63	1.00; 1.06
Processes									-0.07	-0.16; 0.03	.15	0.92; 0.96	-0.07	-0.20; 0.06	.25	0.90; 0.97
People and partners									0.04	-0.12; 0.20	.64	1.00; 1.07	0.17	0.02; 0.33	**.03**	1.16; 1.22
Problem-solving									0.03	-0.12; 0.18	.69	1.00; 1.06	0.04	-0.16; 0.24	.68	0.98; 1.10
Neck complaints at T1													2.40	1.74; 3.03	**<.01**	10.40; 11.40
Female gender	0.84	0.02; 1.66	**.04**	2.28; 2.35	0.52	-0.12; 1.15	.11	1.58; 1.81	0.84	0.03; 1.66	**.04**	2.30; 2.39	0.49	-0.20; 1.18	.17	1.48; 1.76
BMI	-0.01	-0.07; 0.04	.67	0.98; 1.00	-0.03	-0.09; 0.04	.39	0.96; 1.00	0.003	-0.05; 0.06	.90	0.99; 1.02	-0.01	-0.08; 0.06	.76	0.97; 1.01
Age 43 to 51 years	0.16	-0.58; 0.89	.674	1.08; 1.23	0.29	-0.63; 1.21	.54	1.19; 1.43	0.13	-0.61; 0.86	.74	1.08; 1.18	0.26	-0.68; 1.19	.59	1.17; 1.39
52 to 57 years	-0.03	-0.80; 0.75	.95	0.90; 1.07	0.57	-0.39; 1.53	.25	1.60; 1.86	-0.12	-0.92; 0.69	.78	0.79; 0.99	0.44	-0.58; 1.46	.39	1.34; 1.71
58 years and older	-0.54	-1.22; 0.14	.12	0.56; 0.63	-.0.04	-0.82; 0.74	.92	0.87; 1.12	-0.61	-1.32; 0.10	.09	0.51; 0.59	-0.10	-0.91; 0.71	.80	0.79; 1.06

Reference groups in the control variables were male and age ≤ 42; β, unstandardized beta coefficient; T1, time 1; T2 time, 2; 4P, Philosophy, Processes, People and partners, and Problem-solving; CI, confidence interval; P, p-value; OR, odds ratio; MI, multiple imputation.

For peer review only - http://bmjopen.bmj.com/site/about/guidelines.xhtml

Table 8 Association between Lean maturity and musculoskeletal complaints in the low back over time. Predictors were Lean maturity in total at T1 (models 2a and 2b) or each of 4P's (model 2c and 2d); complaints at T2 were the outcome in all models. Gender, BMI and age were included as covariates in all models.

**Low back complaints at T2**	**Model 2a, only Lean maturity in total and covariates**				**Model 2b, complaints T1 added**				**Model 2c, 4P (instead of Lean maturity in total)**				**Model 2d, complaints T1 added**			
	β	95% CI	*P*	OR range; MI data set 1-5	β	95% CI	*P*	OR range; MI data set 1-5	β	95% CI	*P*	OR range; MI data set 1-5	β	95% CI	*P*	OR range; MI data set 1-5
Intercept				-1.34				-1.89				-1.60				-2.20
Lean maturity in total at T1	0.004	-0.02; 0.03	.74	1.00; 1.01	0.02	-0.01; 0.04	.26	1.01; 1.02								
Low back complaints at T1					2.00	1.44; 2.55	**<.01**	6.46; 8.64								
Philosophy									0.11	-0.03; 0.25	.13	1.09; 1.17	0.10	-0.07; 0.27	.25	1.07; 1.18
Processes									-0.05	-0.14; 0.04	.25	0.93; 0.97	-0.07	-0.19; 0.05	.24	0.90; 0.97
People and partners									0.06	-0.15; 0.26	.59	1.02; 1.16	0.15	-0.09; 0.39	.21	1.11; 1.28
Problem-solving									-0.02	-0.17; 0.13	.77	0.95; 1.00	0.001	-0.17; 0.17	.99	0.97; 1.03
Complaints at T1													2.07	1.50; 2.64	**<.01**	6.77; 9.43
Female gender	0.53	-0.32; 1.37	.22	1.51; 1.92	0.50	-0.51; 1.50	.33	1.37; 2.07	0.48	-0.37; 1.33	.27	1.46; 1.81	0.46	-0.57; 1.48	.38	1.28; 2.00
BMI	0.02	-0.05; 0.10	.53	1.01; 1.04	-0.01	-0.09; 0.07	.74	0.97; 1.00	0.04	-0.04; 0.11	.31	1.03; 1.05	0.004	-0.07; 0.08	.91	0.99; 1.02
Age 43 to 51 years	0.38	-0.28; 1.03	.26	1.36; 1.54	0.26	-0.40; 0.92	.44	1.25; 1.36	0.37	-0.32; 1.05	.29	1.36 1.52	0.24	-0.47; 0.94	.51	1.23; 1.32
52 to 57 years	0.18	-0.61; 0.96	.66	1.15; 1.25	0.38	-0.41; 1.17	.35	1.35; 1.76	0.13	-0.71; 0.97	.76	1.08; 1.25	0.28	-0.61; 1.18	.53	1.16 1.67
58 years and older	-0.13	-0.72; 0.46	.66	0.81; 0.96	-0.04	-0.68; 0.60	.91	0.88; 1.09	-0.17	-0.79; 0.44	.58	0. 78; 0.94	-0.08	-0.79; 0.63	.82	0.80; 1.04

Reference groups in the control variables were male and age ≤ 42; β, unstandardized beta coefficient; T1, time 1; T2 time, 2; 4P, Philosophy, Processes, People and partners, and Problem-solving; CI, confidence interval; P, p-value; OR, odds ratio; MI, multiple imputation.

Table 9 Association between Lean maturity and musculoskeletal complaints in the upper back over time. Predictors were Lean maturity in total at T1 (models 2a and 2b) or each of 4P's (model 2c and 2d); complaints at T2 were the outcome in all models. Gender, BMI and age were included as covariates in all models.

**Upper back complaints at T2**	**Model 2a, only Lean maturity in total and covariates**				**Model 2b, complaints T1 added**				**Model 2c, 4P (instead of Lean maturity in total)**				**Model 2d, complaints T1 added**			
	β	95% CI	*P*	OR range; MI data set 1-5	β	95% CI	*P*	OR range; MI data set 1-5	β	95% CI	*P*	OR range; MI data set 1-5	β	95% CI	*P*	OR range; MI data set 1-5
Intercept				-0.74				-0.91				-0.82				-0.94
Lean maturity in total at T1	0.002	-0.02; 0.02	.88	1.00; 1.01	0.003	-0.02; 0.03	.84	1.00; 1.01								
Upper back complaints at T1					2.17	1.43; 2.91	**<.01**	7.44; 9.50								
Philosophy									0.01	-0.14; 0.16	.86	0.95; 1.08	0.008	-0.16; 0.18	.93	0.96; 1.08
Processes									-0.04	-0.14; 0.06	.40	0.94; 0.98	-0.07	-0.20; 0.07	.32	0.90; 0.98
People and partners									0.13	-0.10; 0.36	.25	1.09; 1.24	0.15	-0.12; 0.42	.26	1.07; 1.32
Problem-solving									-0.03	-0.21; 0.15	.78	0.95; 0.99	0.01	-0.20; 0.23	.89	0.98; 1.06
Complaints at T1													2.21	1.42; 3.01	**<.01**	7.57; 10.56
Female gender	-0.004	-0.75; 0.74	.99	0.82; 1.15	-0.41	-1.39; 0.57	.41	0.53; 0.78	-0.05	-0.79; 0.70	.90	0.78; 1.11	-0.44	-1.42; 0.55	.38	0.52; 0.77
BMI	0.002	-0.07; 0.07	.96	0.99; 1.03	-0.02	-0.10; 0.066	.71	0.96; 1.01	0.01	-0.07; 0.09	.79	0.99; 1.04	-0.01	-0.10; 0.09	.91	0.97; 1.03
Age 43 to 51 years	0.14	-0.59; 0.87	.70	0.96; 1.39	0.32	-0.55; 1.19	.46	1.04; 1.84	0.15	-0.62; 0.92	.69	0.97; 1.42	0.34	-0.58; 1.26	.46	1.05; 1.85
52 to 57 years	-0.27	-1.13; 0.59	.54	0.72; 0.86	-0.29	-1.38; 0.81	.61	0.65; 0.85	-0.31	-1.19; 0.57	.49	0.67; 0.84	-0.36	-1.53; 0.81	.55	0.59; 0.85
58 years and older	-0.65	-1.35; 0.05	.07	0.47; 0.59	-0.57	-1.38; 0.24	.17	0.49; 0.68	-0.67	-1.39; 0.05	.07	0.46; 0.58	-0.60	-1.47; 0.27	.18	0.46; 0.68

Reference groups in the control variables were male and age ≤ 42; β, unstandardized beta coefficient; T1, time 1; T2 time, 2; 4P, Philosophy, Processes, People and partners, and Problem-solving; CI, confidence interval; P, p-value; OR, odds ratio; MI, multiple imputation.

For peer review only - http://bmjopen.bmj.com/site/about/guidelines.xhtml

DISCUSSION

The prevalence of musculoskeletal complaints reported in the current study is higher than, or similar to the prevalence found in previous studies among hospital staff(21,24, 36). In two other studies in healthcare, if not from Sweden, the prevalence of low back pain during a 12-month period was higher than in the present study(24,26,37), whereas the prevalence of shoulder complaints was lower(21,24). The study by Ribeiro et al.(26) from primary care in Portugal reported a 63% prevalence of low back complaints. In a study of different occupations in Iran, the prevalence of low back pain among healthcare staff was 58%(37), i.e. larger than in our sample. Of particular note, we found that 6% to 9% of the participants had daily musculoskeletal complaints, and that 3% to 7% had been forced to change work tasks or job due to their complaints. A history of pain is strongly associated with current pain(38,39), and studies – however, mainly among blue-collar workers – have suggested uninterrupted, severe neck and shoulder pain over a long period of time to be associated with decreased workability and increased sick leave(40).

We did not find any general association between Lean maturity and musculoskeletal complaints. However, cross-sectional analyses of each of the 4P showed that Philosophy was positively associated with neck complaints, while People and partners had a negative association. In the longitudinal analysis, none of the 4P were associated with complaints in any of the four investigated body regions, except for People and partners, which was positively associated with neck complaints when the other 4P were controlled for and complaints at T1 was included as a covariate. These results are to some extent difficult to interpret, but they can be explained by earlier findings suggesting that both physical and psychosocial working conditions, i.e. factors that we did not assess but which can be influenced by Lean maturity, are risk factors for musculoskeletal complaints(17,31). Since

For peer review only

few studies have been conducted in primary care, we cannot compare our results to previous reports from that sector, and it is of less relevance to compare our result with studies conducted in a hospital setting due to major differences in both physical and psychosocial working conditions between working in hospital (20,22,24,25,26–29) and primary care(31).

Also, our results differ from studies of other occupational sectors than healthcare, where increased Lean has been identified as a risk factor for musculoskeletal complaints(17-19). Likely, the effect of Lean depends on occupational sector, inasmuch as both working conditions and implementations of Lean will differ according to the occupational context. Thus, our results are difficult to compare with previous studies because they may have used other descriptions of Lean, and have investigated other ways of implementing Lean than those pertinent to primary care(1,4). We measured Lean maturity using the LiHcQ, which addresses all Liker's 14 Lean principles(5), while most of the studies included in Mazzocato et al.'s(10) review of Lean in healthcare assessed Lean to a less comprehensive level. Another important aspect in the context of previous papers is the risk of publication bias, i.e. that published studies mainly report significant results, while leaving out non-significant results(41). Our results indicate that musculoskeletal complaints among primary care staff are a concern, especially as some staff have daily complaints and some have to change jobs or work tasks due to these complaints.

We did not find any clear associations between Lean maturity and musculoskeletal complaints, but we encourage future research to assess both Lean maturity and musculoskeletal complaints over a more extended period of time than one year. Further research will allow for a deeper understanding of whether changes in Lean maturity and musculoskeletal complaints are associated, and if so, what elements of Lean that are particularly important risk factors.

For peer review only

STRENGTHS AND LIMITATIONS

One limitation of the study is the use of a convenience sample, and the relatively low response rate(39); this limits the generalizability of our results. Using staff reports of Lean maturity and musculoskeletal complaints can also be viewed as a limitation, as responses may suffer from common-method bias related to the participants' attitudes toward their workplace, their general health, their tendency to give socially desirable answers, and inaccurate recall. On the other hand, staff members can be considered to have the best insights into their own health and working conditions. Our sample comprised mainly female registered nurses. However, this reflected well the composition of primary care staff in Sweden, where the most common profession is registered nurses(42), and even healthcare in general, where 88% of the registered nurses are women(43). Data concerning activities conducted by the primary care units to adopt Lean between T1 and T2 were not collected, which limits the interpretation of our results. A strength of the study was its longitudinal design, and the use of valid and, in general, consistent instruments for measuring Lean maturity(33) as well as complaints. An additional strength was the MI approach for handling missing data, since this procedure does not lead to loss of data, such as several other alternatives. However, a major limitation of our study is the follow-up time frame of only one year. It may well be too short to pick up changes in musculoskeletal health, which may occur at slower rate, and changes in Lean, which may be small during a period of one year (figure 2).

CONCLUSIONS

The prevalence of musculoskeletal complaints among primary care staff was high and did not change during one year. The extent of Lean maturity was not associated with complaints, neither in cross-sectional analyses nor in a one-year predictive analysis.

For peer review only

Acknowledgement We would like to thank all participants in the study, and their primary health care institutions.

Funding This research was financially supported by the University of Gävle. No grant number.

Disclaimer Funders had no role in the study design, data collection, analysis, data interpretation or the decision to submit the paper for publication.

Competing interests None declared.

Author contribution The study was designed by MK, SEM, LB, HH and ME. Data were collected by MK and analyzed by MK, HH and ME. MK was primarily responsible for drafting the manuscript, which was critically revised by SEM, LB, HH and ME. All authors, i.e. MK, SEM, LB, HH and ME, read and approved the final manuscript.

Patient consent Patients were not involved in the research.

Ethical approval The study was approved by the Regional Ethical Review Board in Uppsala (Reg. no. 2014/525 and 2014/525/1).

Provenance and peer review Not commissioned; externally peer reviewed.

Data sharing statement The data presented in this study are available from the corresponding author on reasonable request. Individual data are not publicly available due to general data protection regulations (GDPR), and personal data ordinance (PDPO).

Open access This is an open access article distributed in accordance with the Creative Commons Attribution Non Commercial (CC BY-NC 4.0) license, which permits others to distribute, remix, adapt, build upon this work non-commercially, and license their derivative works on different terms, provided the original work is properly cited, appropriate credit is given, any changes made indicated, and the use is non-commercial. See:http://creativecommons.org/licenses/by-nc/4.0/.

For peer review only

REFERENCES

1. D'Andreamatteo A, Ianni L, Lega F, Sargiacomo M. Lean in healthcare: A comprehensive review. *Health Policy*. 2014;119(9).

2. Holden RJ. Lean Thinking in emergency departments: a critical review. *Ann Emerg Med*. 2011;57(3):265–78.

3. Joosten T, Bongers I, Janssen R. Application of lean thinking to health care: issues and observations. *Int J Qual Health Care.* 2009;21(5):341–7.

4. Souza LB De. Trends and approaches in lean healthcare. *Leadersh Heal Serv.* 2009;22(2):121–39.

5. Liker JK. The Toyota Way. New York: McGraw-Hill; 2004.

6. Pettersen J. Defining lean production: some conceptual and practical issues. *TQM J.* 2009;21(2):127–42.

7. Langstrand J, Drotz E. The rhetoric and reality of Lean: a multiple case study. *Total Qual Manag Bus Excell.* 2015;:1–15.

8. Kaltenbrunner M, Mathiassen SE, Bengtsson L, et al. Lean maturity and quality in primary care. *J Health Organ Manag.* 2019;33(2):141–154

9. Axelsson, R. (2000). "The organizational pendulum: Healthcare management in Sweden 1865-1998." Scandinavian Journal of Public Health **28**(1): 47-53.

10. Mazzocato P, Savage C, Brommels M, et al. Lean thinking in healthcare: a realist review of the literature. *Qual Saf Heal Care.* 2010;25;19(5):376–382.

11. Mazzocato, P., Holden, R. J., Brommels, M., Aronsson, H., Bäckman, U., Elg, M., & Thor, J. (2012). How does lean work in emergency care? A case study of a lean-inspired intervention at the Astrid Lindgren Children's hospital, Stockholm, Sweden. BMC health services research, 12(1), 1-13.

12. Dellve L, Williamsson A, Strömgren M, et al. Lean implementation at different levels in Swedish hospitals: the importance for working conditions and stress. *Int J Hum Factors Ergon.* 2015;3(3–4):235–53.

13. Simons P, Backes H, Bergs J, et al. The effects of a lean transition on process times, patients and employees. *Int J Health Care Qual Assur.* 2017;30(2):103–18.

14. Jorma T, Tiirinki H, Bloigu R, et al. LEAN thinking in Finnish healthcare. *Leadersh Health Serv.* 2016;29(1).

15. Rees GH, Gauld R. Can lean contribute to work intensification in healthcare? *J Health Organ Manag.* 2017;31(3):369–84.

16. Arlbjørn JS, Freytag PV. Evidence of lean: a review of international peer-reviewed journal articles. *Eur Bus Rev.* 2013;25(2):174–205.

For peer review only

17. Koukoulaki T. The impact of lean production on musculoskeletal and psychosocial risks: An examination of sociotechnical trends over 20 years. *Appl Ergon.* 2014;45(2):198–212.

18. Landsbergis PA, Cahill J, Schnall P. The impact of lean production and related new systems of work organization on worker health. *J Occup Health Psychol.* 1999;4(2):108.

19. Brännmark M, Håkansson M. Lean production and work-related musculoskeletal disorders: overviews of international and Swedish studies. *Work.* 2012;41 Suppl 1:2321–8.

20. Kuorinka I, Jonsson B, Kilbom A, et al. Standardised Nordic questionnaires for the analysis of musculoskeletal symptoms. *Appl Ergon.* 1987;18(3):233–7.

21. Long MH, Johnston V, Bogossian F. Work-related upper quadrant musculoskeletal disorders in midwives, nurses and physicians: A systematic review of risk factors and functional consequences. *Appl Ergon.* 2012;43(3):455–67.

22. Sirisawasd S, Taptagaporn S, Boonshuyar C, et al. Interventions commonly used to prevent work-related musculoskeletal disorders among healthcare workers. *J Heal Res.* 2018;32(5):371–83.

23. Saberipour B, Ghanbari S, Zarea K, et al. Investigating prevalence of musculoskeletal disorders among Iranian nurses: A systematic review and meta-analysis. *Clin Epidemiol Glob Heal.* 2018;(7):513–8

24. Freimann T, Pääsuke M, Merisalu E. Work-related psychosocial factors and mental health problems associated with musculoskeletal pain in nurses: a cross-sectional study. *Pain Res Manag.* 2016.

25. Statens offentliga utredningar. Kommunaliserad hemsjukvård, SOU 2011:55 Available from https://www.regeringen.se/49b6a1/contentassets/1bb6a8565d0c40959035a1fa8106ef7b/ko mmunaliserad-hemsjukvard-sou-201155, accessed 09 April 2019.

26. Ribeiro T, Serranheira F, Loureiro H. Work related musculoskeletal disorders in primary health care nurses. *Appl Nurs Res.* 2017;33:72–7.

27. Courvoisierl DS, Genevayl S, Cedraschil C, et al. Job strain, work characteristics and back pain: a study in a university hospital. *Eur J Pain.* 2011;15(6):634–40.

28. Bongers PM, Ijmker S, Van den Heuvel S, et al. Epidemiology of work related neck and upper limb problems: psychosocial and personal risk factors (part I) and effective interventions from a bio behavioural perspective (part II). *J Occup Rehabil.* 2006;16(3):272–95.

29. Feng B, Liang Q, Wang Y, et al. Prevalence of work-related musculoskeletal symptoms of the neck and upper extremity among dentists in China. *BMJ Open.* 2014;4(12):e006451.

30. da Costa BR, Vieira ER. Risk factors for work-related musculoskeletal disorders: a systematic review of recent longitudinal studies. *Am J Ind Med.* 2010;53(3):285–323.

31. Jatobá A, Bellas HC, Bonfatti R, et al. Designing for patient risk assessment in primary health care: a case study for ergonomic work analysis. *Cogn Technol Work.* 2016;18(1):215–31.

32. Hauke, A., Flintrop, J., Brun, E., & Rugulies, R. (2011). The impact of work-related psychosocial stressors on the onset of musculoskeletal disorders in specific body regions: A review and meta-analysis of 54 longitudinal studies. Work & Stress, 25(3), 243-256.

For peer review only

33. Kaltenbrunner M, Bengtsson L, Mathiassen SE, et al. A questionnaire measuring staff perceptions of Lean adoption in healthcare: development and psychometric testing. *BMC Health Serv Res.* 2017;17(1):235.

34. Kaltenbrunner M, Bengtsson L, Mathiassen SE, et al. Staff perception of Lean, care-giving, thriving and exhaustion: a longitudinal study in primary care. *BMC Health Serv Res.* 2019;19(1):1–10.

35. Newman DA. Missing Data: Five Practical Guidelines. *Organ Res Methods.* 2014;17(4):372–411.

36. Wahlstedt K, Norbäck D, Wieslander G, et al. Psychosocial and ergonomic factors, and their relation to musculoskeletal complaints in the Swedish workforce. *Int J Occup Saf Ergon.* 2010;16(3):311–21.

37. Mehrdad R, Shams-Hosseini NS, Aghdaei S, et al. Prevalence of low back pain in health care workers and comparison with other occupational categories in Iran: A systematic review. *Iran J Med Sci.* 2016;41(6):467.

38. Panken G, Hoekstra T, Verhagen A, et al. Predicting chronic low-back pain based on pain trajectories in patients in an occupational setting: an exploratory analysis. *Scand J Work Environ Health.* 2016;42(6):520–7.

39. Smedley J, Inskip H, Trevelyan F, et al. Risk factors for incident neck and shoulder pain in hospital nurses. *Occup Environ Med.* 2003;60(11):864–9.

40. Hallman DM, Holtermann A, Dencker-Larsen S, et al. Are trajectories of neck–shoulder pain associated with sick leave and work ability in workers? A 1year prospective study. *BMJ Open.* 2019;9(3):e022006.

41. Baruch Y, Holtom BC. Survey response rate levels and trends in organizational research. *Hum relations.* 2008;61(8):1139–60.

42. Swedish Association of Local Authorities and Regions (SALAR). Professions in primary care. https://skl.se/ekonomijuridikstatistik/statistik/personalstatistik/personalenisiffror/tabellerla n dstingsanstalldpersonal2018.27838.html, accessed 08 September 2017.

43. The National Board of Health and Welfare. Statistics on Licensed Health Care Personnel 2016 and Workforce Status 2015. http://www.socialstyrelsen.se/publikationer2018/2018-210,accessed 08 September 2017.

For peer review only

Figure 1 Flowchart showing staff members eligible for the present study, and the flow of responding staff members between T1 and T2. T1, baseline (2015); T2, follow-up (2016).

Figure 2 Changes in individual ratings of Lean maturity between T1 and T2 for non-imputed data (n=104). The value 0 indicates no change in rating between T1 and T2. Negative and positive values show a decrease and an increase, respectively, in Lean maturity from T1 to T2.

For peer review only

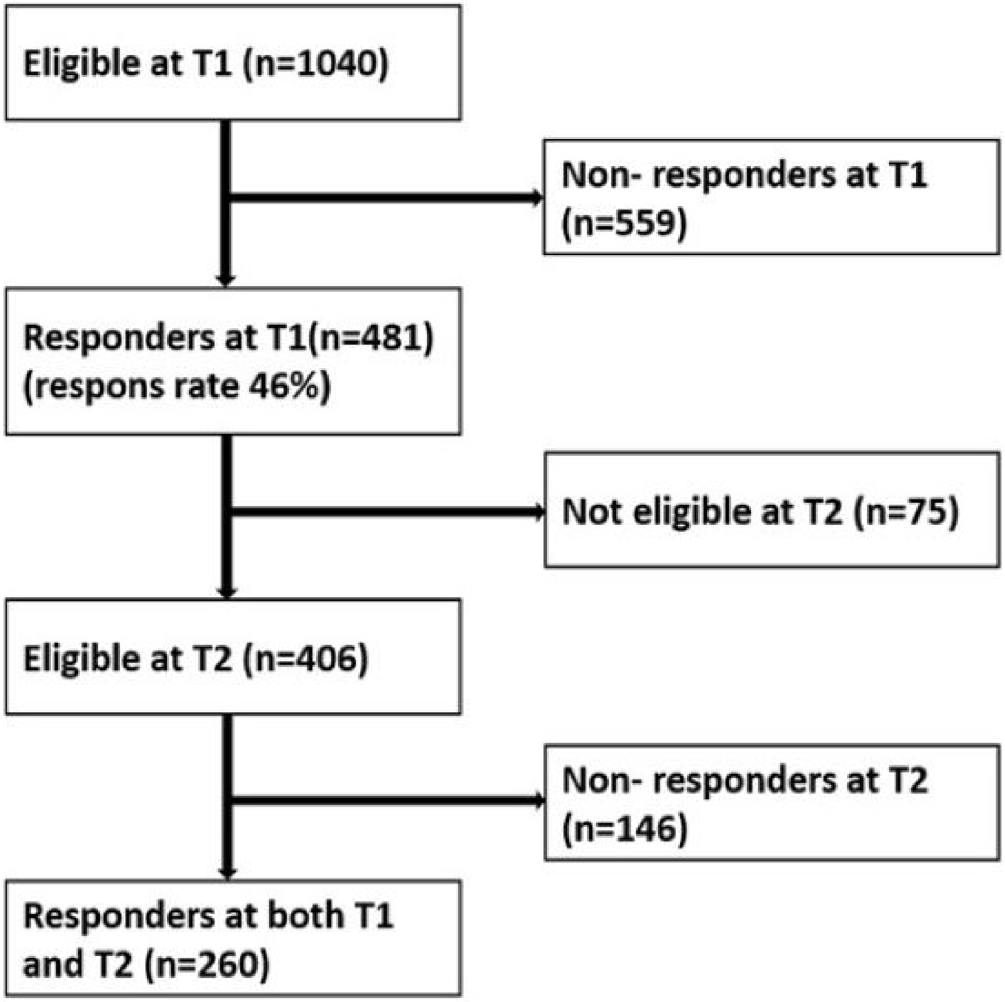

Figure 1 Flowchart showing staff members eligible for the present study, and the flow of responding staff members between T1 and T2. T1, baseline (2015); T2, follow-up (2016).

46x46mm (300 x 300 DPI)

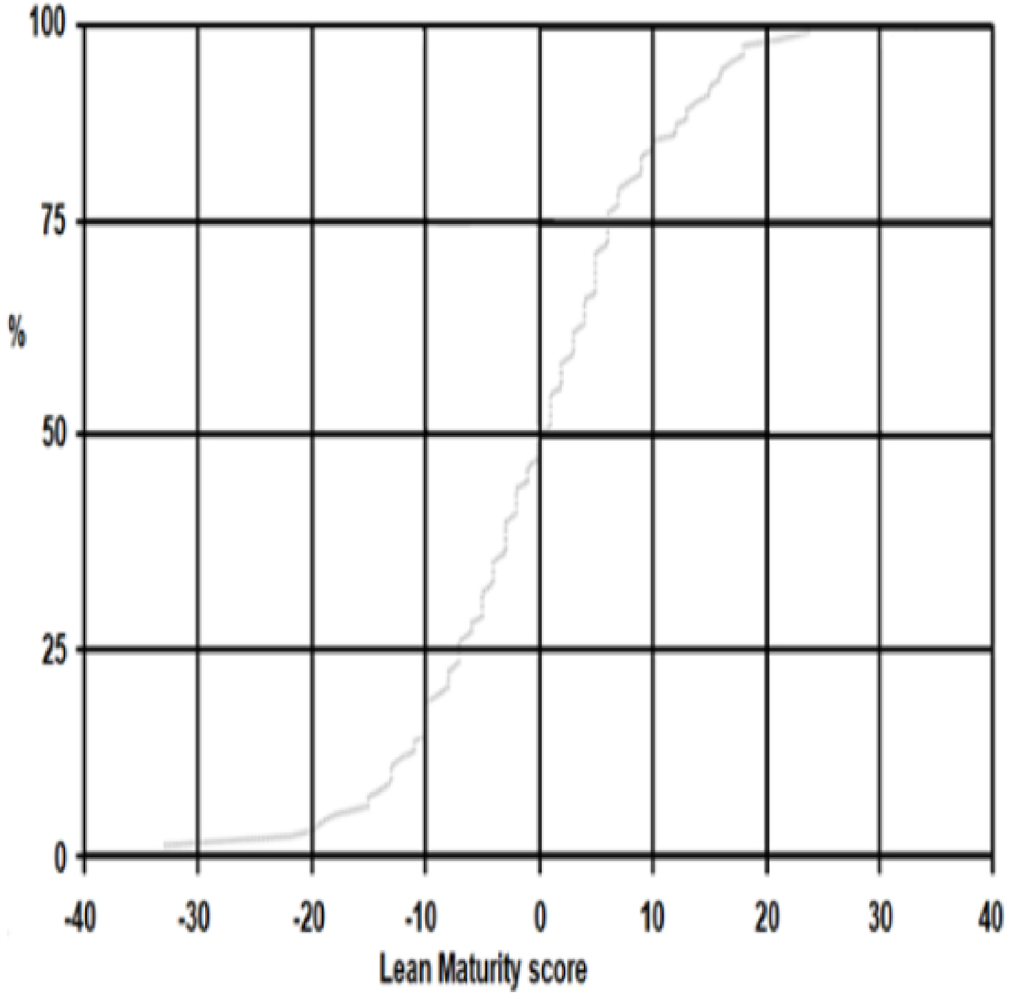

Figure 2 Changes in individual ratings of Lean maturity between T1 and T2 for non-imputed data (n=104). The value 0 indicates no change in rating between T1 and T2. Negative and positive values show a decrease and an increase, respectively, in Lean maturity from T1 to T2.

118x118mm (118 x 118 DPI)

STROBE Statement—Checklist

Referring to the draft received from BMJ open.

Titel: Associations between Lean Maturity in primary care and musculoskeletal complaints among staff. A longitudinal study.

	Item No	Recommendation	Page No	Row
**Title and abstract**	1	(*a*) Indicate the study's design with a commonly used term in the title or the abstract	2	5
		(*b*) Provide in the abstract an informative and balanced summary of what was done and what was found	3,<br>4	5-49<br>3-6
**Introduction**				
Background/rationale	2	Explain the scientific background and rationale for the investigation being reported	5,<br>6,<br>7,<br>8	6-50<br>3-50<br>3-50<br>3-15
Objectives	3	State specific objectives, including any prespecified hypotheses	8 (aim)	8-15
**Methods**				
Study design	4	Present key elements of study design early in the paper	8	24
Setting	5	Describe the setting, locations, and relevant dates, including periods of recruitment, exposure, follow-up, and data collection	8,<br>9	24-50<br>3-33
Participants	6	(*a*) Give the eligibility criteria, and the sources and methods of selection of participants	8,<br>9	45-50<br>3-8, fig 1
Variables	7	Clearly define all outcomes, exposures, predictors, potential confounders, and effect modifiers. Give diagnostic criteria, if applicable	9,<br>10	40-50<br>3-41
Data sources/ measurement	8*	For each variable of interest, give sources of data and details of methods of assessment (measurement). Describe comparability of assessment methods if there is more than one group	9,<br>10	40-50<br>3-41
Bias	9	Describe any efforts to address potential sources of bias	19-20	15-38
Study size	10	Explain how the study size was arrived at		
Quantitative variables	11	Explain how quantitative variables were handled in the analyses. If applicable, describe which groupings were chosen and why	10,<br>11,	47-50<br>3-29, Table1
Statistical methods	12	(*a*) Describe all statistical methods, including those used to control for confounding	11,<br>12	3-29, Table1<br>6-21
		(*b*) Describe any methods used to examine subgroups and interactions	10,<br>11,	50<br>3-29 Table 1
		(*c*) Explain how missing data were addressed	12	6-21
		(*d*) If applicable, describe analytical methods taking account of sampling strategy		
		(*e*) Describe any sensitivity analyses		
**Results**				

For peer review only

Participants	13*	(a) Report numbers of individuals at each stage of study—eg numbers potentially eligible, examined for eligibility, confirmed eligible, included in the study, completing follow-up, and analysed	34	Figure 1
		(b) Give reasons for non-participation at each stage	34	
		(c) Consider use of a flow diagram	34	
Descriptive data	14*	(a) Give characteristics of study participants (eg demographic, clinical, social) and information on exposures and potential confounders	13	Table 2
		(b) Indicate number of participants with missing data for each variable of interest		
Outcome data	15*	Report numbers of outcome events or summary measures	11	15-20
Main results	16	(*a*) Give unadjusted estimates and, if applicable, confounder-adjusted estimates and their precision (eg, 95% confidence interval). Make clear which confounders were adjusted for and why they were included	16-17	Table 5-9
		(*b*) Report category boundaries when continuous variables were categorized		
		(*c*) If relevant, consider translating estimates of relative risk into absolute risk for a meaningful time period		
Other analyses	17	Report other analyses done—eg analyses of subgroups and interactions, and sensitivity analyses	11	3-27
**Discussion**				
Key results	18	Summarise key results with reference to study objectives	17, 18, 19	29-51 3-50 3-38
Limitations	19	Discuss limitations of the study, taking into account sources of potential bias or imprecision. Discuss both direction and magnitude of any potential bias	19, 20	44-51 3-24
Interpretation	20	Give a cautious overall interpretation of results considering objectives, limitations, multiplicity of analyses, results from similar studies, and other relevant evidence	17, 18, 19, 20	30-51 3-50 3-38 3-24
Generalisability	21	Discuss the generalisability (external validity) of the study results	18, 19	38-60 1-15, 44-46
**Other information**				
Funding	22	Give the source of funding and the role of the funders for the present study and, if applicable, for the original study on which the present article is based	21	7-8

For peer review only - http://bmjopen.bmj.com/site/about/guidelines.xhtml

BMJ Open

Associations between Lean maturity in primary care and musculoskeletal complaints among staff: a longitudinal study

Journal:	*BMJ Open*
Manuscript ID	bmjopen-2022-067753.R1
Article Type:	Original research
Date Submitted by the Author:	16-Dec-2022

Complete List of Authors:	Kaltenbrunner, Monica; University of Gävle, Department of Occupational Health Sciences and Psychology<br>Kaltenbrunner, Monica; Linnaeus University Faculty of Health and Life Sciences, Department of Health and Caring Sciences<br>Mathiassen, Svend ; University of Gavle,<br>Bengtsson, Lars; University of Gävle, Department of Industrial Management<br>Högberg, Hans; University of Gävle, Department of Public Health and Caring Sciences<br>Engström, Maria; University of Gävle Faculty of Health and Occupational Studies, Faculty of Health and Occupational Studies; Uppsala University, Department of Public Health and Caring Sciences

**Primary Subject Heading**:	Health services research
Secondary Subject Heading:	Nursing, Health services research
Keywords:	PRIMARY CARE, Musculoskeletal disorders < ORTHOPAEDIC & TRAUMA SURGERY, Health & safety < HEALTH SERVICES ADMINISTRATION & MANAGEMENT, Human resource management < HEALTH SERVICES ADMINISTRATION & MANAGEMENT, Organisational development < HEALTH SERVICES ADMINISTRATION & MANAGEMENT

SCHOLARONE™
Manuscripts

I, the Submitting Author has the right to grant and does grant on behalf of all authors of the Work (as defined in the below author licence), an exclusive licence and/or a non-exclusive licence for contributions from authors who are: i) UK Crown employees; ii) where BMJ has agreed a CC-BY licence shall apply, and/or iii) in accordance with the terms applicable for US Federal Government officers or employees acting as part of their official duties; on a worldwide, perpetual, irrevocable, royalty-free basis to BMJ Publishing Group Ltd ("BMJ") its licensees and where the relevant Journal is co-owned by BMJ to the co-owners of the Journal, to publish the Work in this journal and any other BMJ products and to exploit all rights, as set out in our licence.

The Submitting Author accepts and understands that any supply made under these terms is made by BMJ to the Submitting Author unless you are acting as an employee on behalf of your employer or a postgraduate student of an affiliated institution which is paying any applicable article publishing charge ("APC") for Open Access articles. Where the Submitting Author wishes to make the Work available on an Open Access basis (and intends to pay the relevant APC), the terms of reuse of such Open Access shall be governed by a Creative Commons licence – details of these licences and which Creative Commons licence will apply to this Work are set out in our licence referred to above.

Other than as permitted in any relevant BMJ Author's Self Archiving Policies, I confirm this Work has not been accepted for publication elsewhere, is not being considered for publication elsewhere and does not duplicate material already published. I confirm all authors consent to publication of this Work and authorise the granting of this licence.

For peer review only

Associations between Lean maturity in primary care and musculoskeletal complaints among staff: a longitudinal study

Monica Kaltenbrunner[1]RN, PhD, Svend Erik Mathiassen[1] PhD, Lars Bengtsson[2] PhD, Hans Högberg[3] PhD, Maria Engström[3] RN, PhD

[1]Centre for Musculoskeletal Research, Department of Occupational Health Sciences and Psychology, Faculty of Health and Occupational Studies, University of Gävle, Gävle, Sweden.

[2]Department of Industrial Management, Industrial Design and Mechanical Engineering, Faculty of Engineering and Sustainable Development, University of Gävle, Gävle, Sweden.

[3]Department of Caring Science, Faculty of Health and Occupational Studies, University of Gävle, Gävle, Sweden.

Correspondence to:

Monica Kaltenbrunner

monica.kaltenbrunner.nykvist@hig.se

For peer review only

ABSTRACT

Objective This study had two aims: 1) to determine the prevalence of musculoskeletal complaints among staff in primary care 2) to determine to what extent Lean maturity of the primary care unit can predict musculoskeletal complaints one year later.

Design Descriptive, correlational, and longitudinal design.

Setting Primary care units in mid-Sweden.

Participants In 2015, staff members responded to a web survey addressing Lean maturity and musculoskeletal complaints. The survey was completed by 481 staff members (response rate 46%) at 48 units; 260 staff members at 46 units also completed the survey in 2016.

Outcome measures Associations with musculoskeletal complaints were determined both for Lean maturity in total, and for four Lean domains entered separately in a multivariate model, i.e., Philosophy, Processes, People and partners, and Problem-solving.

Results The shoulders (12-month prevalence 58%), neck (54%), and low back (50%) were the most common sites of 12-months retrospective musculoskeletal complaints at baseline. Shoulders, neck, and low back also showed the most complaints for the preceding 7 days (37%, 33%, and 25%, respectively). The prevalence of complaints was similar at the 1-year follow-up. Total lean maturity in 2015 was not associated with musculoskeletal complaints, neither cross-sectionally nor one year later, for shoulders (one-year β: -0.002, 95% CI -.03 to .02), neck (β: 0.006, 95% CI -.01 to .03), low back (β: 0.004, 95% CI -.02 to .03) and upper back (β: 0.002, 95% CI -.02; .02).

Conclusion The prevalence of musculoskeletal complaints among primary care staff was high and did not change within a year. The extent of Lean maturity at the care unit was not associated with complaints among staff, neither in cross-sectional analyses nor in a one-year predictive analysis.

For peer review only

Keywords: Lean in Health care Questionnaire (LiHcQ), musculoskeletal disorders (MSD), Nordic Musculoskeletal Questionnaire (NMQ), pain

Strengths and limitations of this study

- The longitudinal design allowed analysis over time of associations between Lean maturity and self-reported musculoskeletal complaints.
- The convenience sample and the low response rate are potential limitations.
- Use of self-reports may be a limitation, as responses may be biased by a positive or negative attitude toward Lean and the workplace.

INTRODUCTION

Implementation of Lean has become common in healthcare(1), with an aim to increase efficiency(2) and quality of care(3,4). Lean originates from the automotive industry(5) and has been described and construed in different ways, both there and in healthcare(1,4,6,7).

Several systems for describing Lean have been suggested. Liker's interpretation, which we use in the present study(5), includes 14 basic principles, which can be organized into four categories in a so-called 4P model (Philosophy, Processes, People and partners, and Problem-solving). 'Philosophy' involves having a long-term perspective, focusing on the customer, and having common goals for the staff members to strive for in order to create value for the customer, the organization and society as a whole. 'Processes' involve using resources optimally to improve flow in production. A good flow obtained by, for instance, mapping processes, standardizing work, and reducing waste can increase

For peer review only

quality and efficiency. ‘People and partners’ include showing respect for individuals in the organization and enabling them to grow, as well as respecting suppliers and partners. ‘Problem-solving’ focuses on finding and solving basic causes of problems restraining flow and quality in production(5).

In Lean, staff members are given responsibility and they are expected to contribute to decision-making, and to engage in securing continuous improvements. Customer satisfaction is paramount, and according to Liker, all staff throughout the organization need to be aware of that and contribute to achieving it(5). Liker also claims that all Lean principles must be adopted throughout the organization in order to fully reach the desired results(5). Thus, the ultimate level of Lean maturity will be obtained when all staff adopts all Lean principles to their full extent(8).

In Sweden, Lean was first implemented in industry and began influencing healthcare management in the mid-90s (9). However, several studies in healthcare indicate that Lean has only been partially adopted, with an emphasis on, e.g. standardized work, enhanced seamlessness and flow, and problem-solving(1,4,10,11). Some examples have been reported of positive effects of adopting Lean, including that Lean may improve working conditions(12) and patient safety(13,14). In a previous study in primary care, we showed that Lean maturity was positively association with quality of care as perceived by staff(8). However, other studies conclude that the effects of implementing Lean are ambiguous(15,16). In the context of work-related complaints, Lean should, according to Liker(5), to address human resources in the sense that Lean should not contribute to excessive loads on the staff, leading to negative health consequences, including, e.g. musculoskeletal complaints(17). However, two reviews(17,18) and one overview(19)

For peer review only

suggest that Lean may, indeed, contribute to increased musculoskeletal complaints, at least in other sectors than healthcare. This might be a result of Lean influencing working conditions so that work demands and work pace increase. This, in turn, may lead to decreased opportunities for recovery, which may increase the risk for musculoskeletal complaints(17).

Little is known, however, about associations between Lean and musculoskeletal complaints specifically in healthcare(1,2,10). Musculoskeletal complaints among healthcare staff have been reported to be common(20), and a concern(21). For instance, a review of musculoskeletal complaints among registered nurses, midwives, and physicians working predominantly in hospitals reported that 45% had musculoskeletal complaints in the neck, 40% in the shoulders and 35% in the upper back during a 12-month period, as assessed using the Nordic Musculoskeletal Questionnaire (NMQ)(22). In a cross-sectional study by Freimann et al.(23) among Finnish nurses, the 12-month prevalence of musculoskeletal complaints based on the NMQ was 57% for the low back, 56% for the neck, and 31% for the shoulders.

In healthcare, likely risk factors for musculoskeletal complaints among staff working in hospitals and elderly care are awkward postures, repetitive work, heavy lifting, role conflicts, and high emotional demands together with low influence, lack of respect, and lack of justice(20,23–29). In turn, these risks can be modified by gender, age, and BMI(30). Primary care, however, represents working conditions differing from those in hospitals. In Sweden, primary care offers prevention, advice, and treatment to the citizens and it is the first level of care in the healthcare system(25). Working in primary care has been described as having low physical demands while cognitive demands are

For peer review only

substantial(31). Thus, psychosocial working conditions such as low decision authority, low social support, high job demands, high job strain, and psychological distress - all risk factors for musculoskeletal disorders (MSD)(32) - may prevail in primary care, but may be expressed different than in hospitals. Differences in physical and psychosocial risk factors between primary care and other healthcare areas may lead to differences in complaints and thus different corrective actions.

Few studies have, however, investigated this issue. A cross-sectional study in Portuguese primary care, based on the NMQ, reported that 63% of the participating nurses had low back pain, 50% neck pain, and 41% pain in the dorsal region during the preceding 12-month period(26). In Swedish primary care, the prevalence of musculoskeletal complaints has, to our knowledge, not been assessed in any scientific study. Since Lean sets the scene for working conditions among staff, the association between Lean maturity and musculoskeletal complaints in primary care stands up as an interesting issue for research. Hence, the aim of the present study was twofold: 1) to determine the prevalence of musculoskeletal complaints among staff in primary care and 2) to determine to what extent Lean maturity of the primary care unit could predict musculoskeletal complaints one year later.

METHODS

Primary care units

The study had a descriptive, correlational, and longitudinal design. A convenience sample of 52 primary care units located in a region in mid-Sweden were first approached; 42 units accepted, of which 38 were public primary care units and four belonged to private providers. Due to this shortage of private care units, one of the largest nationwide

For peer review only

private health care providers (organizing 85 primary care units) was approached; six units from there agreed to participate, in addition to the four already recruited. Units were required to, according to their own opinion, have adopted Lean to some extent to be eligible for participation in the study.

Participants

All staff at the recruited units were asked to participate, and responded to a questionnaire in May 2015 (T1) and again in May 2016 (T2), with an inclusion criterion of having worked at the unit at least three months prior to data collection. The number of staff eligible at T1 was 1040; 481 responded, distributed among 48 units, i.e. a 46% response rate. At T2, 406 staff members of those who responded at T1 were still eligible. In total, 260 staff members at 46 units responded at both T1 and T2 (Figure 1).

Data collection procedure

Data were collected at T1 and T2 using a web survey. After two non-successful reminders, non-responders were sent a paper version. Confidentiality and voluntary participation were emphasized at data collection. Consent to participate was given when participants completed and returned the questionnaire. The study is part of a larger research project on Lean maturity in primary care; data have been used and published for other purposes in previous papers(8,33,34).

Measurements

Staff members' self-rated perception of *Lean maturity* at their unit was assessed using the 16-item Lean in Health care Questionnaire (LiHcQ), shown to have acceptable psychometric properties(33). The LiHcQ is based on the 4P model of Lean proposed by

For peer review only

Liker(5), addressing Philosophy (3 items), Processes (7 items), People and partners (3 items), and Problem-solving (3 items). Answers are provided on a scale ranging from 1, indicating low Lean maturity, to 5, indicating high Lean maturity. Thus, the total LiHcQ score could range from 16 to 80. In the present study, Cronbach's alpha was 0.93 for total Lean maturity, and for Philosophy 0.75, Processes 0.86, People and partners 0.60, and Problem-solving 0.81, respectively. *Musculoskeletal complaints* (pain and/or discomfort) were assessed using the Nordic Musculoskeletal Questionnaire, NMQ(24). All 18 items in the first part of the NMQ were included; they address, with response alternatives "yes" and "no", the occurrence of musculoskeletal complaints during the past 12-month and 7-day periods in nine body regions (neck, shoulders, low back, upper back, elbows, hands/wrists, hips, knees and ankles/feet). In the second part of the NMQ (which in the present study addressed pain only in the neck, shoulders, and low back), the respondent is asked to respond with a "yes" or "no" to whether she, during the preceding year, has been limited in performing activities during work or leisure, has changed jobs or work tasks, or has visited a health practitioner due to musculoskeletal complaints. Items also address the total duration of musculoskeletal complaints during the preceding year (5-point scale, with a 1 for 0 days and a 5 for every day). Participants were also asked to report gender, age, height, weight, years worked in the profession, and years worked at the present primary care unit. Lean maturity as well as musculoskeletal complaints were assessed both at T1 and T2.

Statistical analysis

Data analyses were conducted using IBM SPSS Statistics, version 24. Descriptive statistics were used for demographic data, the NMQ and LiHcQ. Differences between T1 and T2 in musculoskeletal complaints were analyzed using McNemar's test; changes in

For peer review only

Lean maturity over time were analyzed using a paired *t*-test. Associations between variables in the cross-sectional as well as the longitudinal case were determined using Generalized estimating equations (GEE), with an exchangeable correlation structure accounting for the correlated nature of data collected at the same primary care unit. Predictive analyses over time were conducted using LiHcQ scores at T1 as the predictor variable, and musculoskeletal complaints at T2 as the outcome, controlling for complaints at T1. Additional control variables were gender, BMI, and age categorized in quartiles. Since musculoskeletal complaints showed to be most prevalent in shoulders, neck, low back and upper back, these body regions were selected for analysis of associations between Lean maturity and complaints. Two models were tested based on cross-sectional data, and four models based on longitudinal data (Table 1).

Table 1. Variables included in the six models addressing associations between Lean maturity and musculoskeletal complaints

	Cross-sectional data		Longitudinal data			
	Model 1a	Model 1b	Model 2a	Model 2b	Model 2c	Model 2d
**Independent variables**						
Lean maturity at T1, in total	X		X	X		
Lean maturity at T1, each of the 4P		X			X	X
**Dependent variables**						
Complaints at T1	X	X		X		X
Complaints at T2			X	X	X	X

All six models included gender, body mass index and age (in quartiles) as control variables.
T1, time 1 (2015); T2, time 2 (2016);
4P: Philosophy, Processes, People and partners, and Problem-solving.

Missing data were handled using multiple imputation (MI; 35). When conducting a MI, five new datasets are produced with imputed data. In the subsequent analysis, results from the original dataset and the five MI datasets are generated, as well as a pooled result. We report the pooled results, in terms of regression coefficients with 95%

For peer review only

confidence intervals (CI) and corresponding p-values. Standard deviations (SD) and odds ratios (OR) were obtained on basis of the five MI datasets, since pooled results do not provide these metrics.

Patient and public involvement

None.

RESULTS

Participants

The sample at T1 consisted mostly of females, their mean age was 50.2 years (SD 10.3) (Table 2). The most common profession was registered nurse, and most of the participants worked in public primary care units. Non-responders at T2, i.e. those only responding at T1, did not differ significantly from those responding at both T1 and T2 with respect to gender, age, years worked in the profession and years worked at the present unit.

Table 2. Participant characteristics

	T1 N:481	(%)	T2 N:260	(%)
**Participants working at**				
Public primary care units	407	(85)	216	(83)
Private primary care units	74	(15)	44	(17)
**Gender**				
Women	422	(88)	224	(86)
Men	59	(12)	35	(13)
**Profession**				
Registered Nurses	181	(38)	101	(39)
Physician	70	(15)	37	(14)
Administrator and secretary	64	(13)	31	(12)
Physiotherapist	47	(10)	30	(12)
Licensed Practical Nurse	45	(9)	24	(9)
Social worker and psychologist	41	(9)	21	(8)
Manager	26	(5)	17	(7)
Occupational therapist	17	(4)	8	(3)

For peer review only

Dietician	3	(1)	1	(< 1)
**Age**<br>Mean (SD)	50.2 (10.3)		50.6 (10.0)	
Md (Q_1-Q_3)	52.0 (44.0-59.0)		53.0 (44.0-59.0)	
**BMI**<br>Mean (SD)	25.0 (4.0)		25.4 (4.1)	
Md (Q_1-Q_3)	24.2 (22.4-27.4)		24.6 (22.6-27.8)	
**Years worked in the profession**<br>Mean (SD)	21.5 (12.1)		22.0 (11.8)	
Md (Q_1-Q_3)	21.0 (11.0-31.0)		20.0 (13.0-32.0)	
**Years worked at the present unit**<br>Mean (SD)	9.1 (9.0)		9.3 (8.7)	
Md (Q_1-Q_3)	5.0 (2.0-14.0)		5.0 (3.0-14.0)	

T1, time 1 (2015); T2, time 2 (2016); BMI, body mass index; Md, median. Numbers regarding professions do not add up to 481 and 260 because some participants had multiple professions.

Prevalence of musculoskeletal complaints

At T1, 50% to 58% of the participants (n=481) reported musculoskeletal complaints in the preceding 12-month period in the shoulder, neck or low back (Table 3). Musculoskeletal complaints were also most common in these three body regions when reported for the preceding seven days (Table 4). The same pattern was found when analyzing only those who responded at both T1 and T2 (n=260).

Table 3. Prevalence of musculoskeletal complaints at T1 (n=481)

	Musculo-skeletal complaints	Musculo-skeletal complaints during the preceding 7-day period	Musculoskeletal complaints limiting activities during…* ..leisure	..work	Changed job or work tasks due to musculo-skeletal complaints *	Visited a health practitioner due to musculo-skeletal complaints *
	n (%)	n (%)	n (%)	n (%)	n (%)	n (%)
Shoulders	264 (58%)	176 (37%)	86 (18%)	30 (6%)	23 (5%)	86 (18%)
Neck	249 (54%)	157 (33%)	77 (16%)	35 (7%)	15 (3%)	75 (16%)
Low back	222 (50%)	119 (25%)	102 (21%)	44 (9%)	33 (7%)	70 (15%)
Upper back	160 (36%)	74 (15%)				
Wrists/hands	133 (30%)	81 (17%)				
One hip or both hips	121 (27%)	72 (15%)				
One knee or both knees	121 (27%)	71 (15%)				
One or both ankles/feet	112 (25%)	76 (16%)				
Elbows	54 (12%)	29 (6%)				

For peer review only

Data concern the preceding 12-month period if not stated otherwise.
*These outcomes were obtained only for shoulders, neck and low back, as explained in the running text.
All percentages refer to n=481.

At T1 (n=481), musculoskeletal complaints during the preceding 12-month period most often lasted for 1-7 days for the low back (21%) and neck (20%), while shoulder complaints most often lasted for more than 30 days (15%). Nine percent reported daily musculoskeletal complaints in the past 12-month period in the shoulders, 7% in the low back, and 6% in the neck.

Changes in musculoskeletal complaints

Changes over time in musculoskeletal complaints were analyzed for shoulders, neck, low back, and upper back. Between 20% and 43% of the participants reported musculoskeletal complaints from these body regions at both T1 and T2 (Table 4). Between 12% and 13% reported musculoskeletal complaints in the four body regions at T1 which were not present anymore at T2. Between 11% and 14% had no musculoskeletal complaints at T1, but reported complaints at T2. Most of the participants (72% to 76%) did not change their ratings between T1 and T2. Differences between T1 and T2 in musculoskeletal complaints were non-significant for all four body regions (Table 4).

Table 4. Combined prevalence of musculoskeletal complaints at T1 and T2, including results of McNemar's test for differences in ratings between T1 and T2

	**Shoulders** (n=260)	**Neck** (n=260)	**Low back** (n=260)	**Upper back** (n=260)
Musculoskeletal complaints at T1 and T2	110 (42%)	113 (43%)	94 (36%)	51 (20%)
Musculoskeletal complaints at T1 but not at T2	33 (13%)	31 (12%)	34 (13%)	32 (12%)
No musculoskeletal complaints at T1 but complaints at T2	37 (14%)	32 (12%)	37 (14%)	29 (11%)

For peer review only

Musculoskeletal complaints neither at T1 nor at T2	80 (31%)	84 (32%)	94 (36%)	147 (57%)
McNemar test, original dataset, P	0.892	0.609	0.775	0.788
MI-dataset 1-5, P range	0.899 to 1.000	0.470 to 0.906	0.366 to 1.000	0.550 to 1.000

T1, time 1; T2, time 2. MI, multiple imputation. P calculated with binomial distribution.

Lean maturity

Mean Lean maturity was 46.2 at T1 (SD ranging from 11.5 to 11.7 in the MI dataset), and 45.3 at T2 (SD ranging from 11.9 to 12.1). Between T1 and T2, 94.5% of all participants changed their rating of total Lean maturity, however these changes were both positive and negative (Figure 2). Thus, no statistically significant difference in Lean maturity was found at the group level between T1 and T2 (pooled data; p=.16).

Associations between Lean maturity and musculoskeletal complaints

Cross-sectional data at T1 showed no statistically significant associations between Lean maturity in total and musculoskeletal complaints in the shoulders, neck, low back or upper back (p between .09 and .96; Table 5, Model 1a). Using each of the 4P as the independent variable instead of Lean maturity in total did not result in any statistically significant associations either, except for neck complaints showing a positive association with Philosophy (p= <.01; Table 5, Model 1b) and a negative association with People and partners (p=.03).

For peer review only

Table 5. Cross-sectional associations between Lean maturity and musculoskeletal complaints in the shoulders, neck, low back and upper back

	Model 1a				Model 1b			
	β	95% CI	*P*	OR range; MI data set 1-5	β	95% CI	*P*	OR range; MI data set 1-5
**Shoulder complaints** Intercept -0.61					Intercept -0.80			
**Lean maturity in total**	-0.02	-0.04; 0.01	.17	0.98; 0.99				
**Philosophy**					0.12	-0.02; 0.26	.09	1.09; 1.17
**Processes**					0.01	-0.13; 0.15	.89	0.97; 1.09

**People and partners**					-0.16	-0.34; 0.03	.10	0.83; 0.89
**Problem-solving**					-0.08	-0.33; 0.17	.49	0.81; 0.98
**Control variables**								
**Female gender**	0.91	0.13; 1.69	**.02**	2.32; 2.62	0.88	0.07; 1.69	**.03**	2.20; 2.59
**BMI**	0.03	-0.05; 0.10	.51	1.02; 1.04	0.03	-0.06; 0.11	.56	1.02; 1.03
**Age 43 to 51 years**	0.20	-0.56; 0.97	.61	1.15; 1.37	0.23	-0.58; 1.04	.58	1.13; 1.43
**52 to 57 years**	0.74	-0.20; 1.67	.12	2.02; 2.14	0.85	-0.16; 1.86	.10	2.13; 2.80
**58 years and older**	-0.22	-0.10; 0.55	.58	0.77; 0.86	-0.22	-1.04; 0.59	.59	0.76; 0.86
**Neck complaints** Intercept -0.34					Intercept -.60			
**Lean maturity in total**	-0.01	-0.03; 0.01	.50	0.99; 1.00				
**Philosophy**					0.19	0.07; 0.31	**<.01**	1.19; 1.23
**Processes**					-0.02	-0.13; 0.08	.66	0.95; 1.00
**People and partners**					-0.19	-0.36; -0.02	.03	0.78; 0.85
**Problem-solving**					-0.00	-0.22; 0.21	.97	0.93; 1.06
**Control variables**								
**Female gender**	1.02	-0.05; 2.09	.06	2.60; 3.05	1.06	-0.07; 2.18	.07	2.51; 3.36
**BMI**	0.02	-0.04; 0.08	.55	1.01; 1.03	0.03	-0.04; 0.09	.41	1.02; 1.04
**Age 43 to 51 years**	-0.07	-0.81; 0.67	.85	0.92; 0.95	-0.12	-0.88; 0.64	.76	0.84; 0.92
**52 to 57 years**	-0.90	-1.65; -0.15	**.02**	0.39; 0.43	-0.94	-1.74; -0.13	**.02**	0.36; 0.46
**58 years and older**	-0.97	-1.77; -0.17	**.02**	0.36; 0.40	-1.05	-1.93; -0.18	**.02**	0.32; 0.37
**Low back complaints** Intercept -1.44					Intercept -1.59			
**Lean maturity in total**	-0.02	-0.04; 0.00	.09	0.98; 0.98				
**Philosophy**					0.08	-0.06; 0.21	.29	1.06; 1.12
**Processes**					0.00	-0.11; 0.12	.97	0.95; 1.04
**People and partners**					-0.14	-0.33; 0.05	.14	0.83; 0.92
**Problem-solving**					-0.05	-0.29; 0.19	.66	0.89; 1.07
**Control variables**								
**Female gender**	0.28	-0.56; 1.12	.51	1.18; 1.66	0.26	-0.65; 1.17	.57	1.08 1.66
**BMI**	0.08	0.01; 0.15	**.02**	1.08; 1.09	0.08	0.01; 0.16	**.03**	1.07; 1.10
**Age 43 to 51 years**	0.39	-0.43; 1.20	.35	1.34; 1.59	0.40	-0.40; 1.20	.33	1.38; 1.55
**52 to 57 years**	-0.28	-1.20; 0.65	.56	0.58; 0.88	-0.21	-1.24; 0.83	.69	0.56; 0.98

**58 years and older**	-0.22	-0.96; 0.52	.56	0.68; 0.91	-0.22	-0.99;	.58	0.67; 0.92
**Upper back complaints** Intercept -1.96					Intercept -2.01			
**Lean maturity in total**	0.00	-0.02; 0.02	.96	1.00; 1.00				
**Philosophy**					.01	-0.11;	.82	0.96; 1.05
**Processes**					.02	-0.09; 0.12	.77	0.98; 1.03
**People and partners**					.04	-0.14; 0.23	.65	1.01; 1.10
**Control variables**								
**Female gender**	0.75	-0.21; 1.70	.13	1.88; 2.34	0.69	-0.29; 1.67	.17	1.78; 2.27
**BMI**	0.03	-0.04; 0.10	.40	1.02; 1.03	0.03	-0.04; 0.10	.44	1.02; 1.04
**Age 43 to 51 years**	-0.25	-0.89; 0.40	.46	0.74; 0.85	-0.22	-0.89; 0.45	.52	0.76; 0.86
**52 to 57 years**	-0.10	-0.99; 0.79	.83	0.83; 1.01	-0.05	-0.98; 0.88	.92	0.87; 1.06
**58 years and older**	-0.43	-1.21; 0.36	.28	0.60; 0.71	-0.40	-1.21; 0.41	.33	0.60; 0.74

Reference in the control variables were male and age ≤ 42. OR, odds ratio; 4P, Philosophy, Processes, People and partners, and Problem-solving; β, unstandardized beta coefficient; CI, confidence interval; MI, multiple imputation; P, p-value; BMI, body mass index.

Similarly, when analyzing longitudinal data, we found no statistically significant associations between Lean maturity in total at T1 and musculoskeletal complaints in the four body regions at T2 (p from .57 to .88) (Table 6, 7, 8 and 9). When analyzing associations between complaints at T2 and each of the individual 4P at T1, adding complaints at T1 as a covariate, People and partners were positively associated with neck complaints (p=.03) (Table 7, model 2d).

Table 6. Association between Lean maturity and musculoskeletal complaints in the shoulders over time

Shoulder complaints at T2	Model 2a, only Lean maturity in total and covariates				Model 2b, complaints T1 added				Model 2c, each of the 4P				Model 2d, Complaints T1 added			
	β	95% CI	*P*	OR range; MI data set 1-5	β	95% CI	*P*	OR range; MI data set 1-5	β	95% CI	*P*	OR range; MI data set 1-5	β	95% CI	*P*	OR range; MI data set 1-5
Intercept				-0.29				-1.02				-0.43				-1.12
Lean maturity in total at T1	-0.002	-0.03; 0.02	.85	0.996; 1.00	0.006	-0.02; 0.03	.61	1.00; 1.10								
Shoulder complaints at T1					2.00	1.36; 2.66	**<.01**	6.83; 8.07								
Philosophy									0.07	-0.05; 0.19	.23	1.06; 1.09	0.03	-0.11; 0.16	.70	1.05; 1.01
Processes									-0.03	-0.13; 0.08	.64	0.95; 1.01	-0.04	-0.14; 0.07	.51	0.95; 1.00
People and partners									-0.01	-0.18; 0.15	.88	0.96; 1.01	0.07	-0.11; 0.25	.46	1.04; 1.10
Problem-solving									-0.02	-0.19; 0.16	.85	0.92; 1.03	0.02	-0.13; 0.17	.80	0.98; 1.06
Complaints at T1													2.02	1.36; 2.68	**<.01**	6.72; 8.22
Female gender	0.29	-0.26; 0.83	.30	1.31; 1.36	-0.14	-0.69; 0.41	.61	0.83; 0.93	0.27	-0.28; 0.82	.34	1.22; 1.36	-0.14	-0.72; 0.43	.62	0.81; 0.96
BMI	0.02	-0.05; 0.08	.63	1.00; 1.03	0.01	-0.06; 0.07	.85	0.99; 1.02	0.02	-0.05; 0.09	.55	1.00; 1.04	0.01	-0.06; 0.08	.69	0.99; 1.03
Age 43 to 51 years	0.03	-0.74; 0.80	.94	0.96; 1.11	-0.08	-0.98; 0.82	.87	0.86; 1.01	0.03	-0.76; 0.82	.942	0.95; 1.09	-0.08	-1.00; 0.83	.86	0.87; 1.00
52 to 57 years	0.34	-0.52; 1.20	.44	1.29; 1.51	0.02	-0.92; 0.96	.96	0.92; 1.12	0.34	-0.56; 1.24	.456	1.24; 1.48	-0.03	-0.99; 0.94	.96	0.86; 1.05
58 years and older	-0.26	-0.94; 0.43	.47	0.73; 0.84	-0.20	-0.86; 0.46	.56	0.76; 0.92	-0.27	-0.98; 0.44	.454	0.71; 0.86	-0.21	-0.87; 0.45	.52	0.76; 0.90

Predictors were Lean maturity in total at T1 (models 2a, 2b), or each of the 4Ps (models 2c, 2d); complaints at T2 were the outcome in all models. Gender, BMI and age were included as covariates in all models. Reference in the control variables were male and age ≤ 42; β, unstandardized beta coefficient; T1, time 1; T2 time, 2; 4P, Philosophy, Processes, People and partners, and Problem-solving; CI, confidence interval; P, p-value; OR, odds ratio; MI, multiple imputation.

For peer review

Table 7. Association between Lean maturity and musculoskeletal complaints in the neck over time

Neck complaints at T2	Model 2a, only Lean maturity in total and covariates				Model 2b, complaints T1 added				Model 2c, 4P (instead of Lean maturity in total)				Model 2d, complaints T1 added			
	β	95% CI	*P*	OR range; MI data set 1-5	β	95% CI	*P*	OR range; MI data set 1-5	β	95% CI	*P*	OR range; MI data set 1-5	β	95% CI	*P*	OR range; MI data set 1-5
Intercept				-0.36				-1.53				-0.60				-1.74
Lean maturity in total at T1	0.006	-0.01; 0.03	.57	1.00; 1.01	0.01	-0.01; 0.04	.28	1.01; 1.02								
Neck complaints at T1					2.31	1.66; 2.96	**<.01**	9.54; 10.71								
Philosophy									0.12	-0.02; 0.25	.08	1.09; 1.15	0.03	-0.10; 0.17	.63	1.00; 1.06
Processes									-0.07	-0.16; 0.03	.15	0.92; 0.96	-0.07	-0.20; 0.06	.25	0.90; 0.97
People and partners									0.04	-0.12; 0.20	.64	1.00; 1.07	0.17	0.02; 0.33	**.03**	1.16; 1.22
Problem-solving									0.03	-0.12; 0.18	.69	1.00; 1.06	0.04	-0.16; 0.24	.68	0.98; 1.10
Neck complaints at T1													2.40	1.74; 3.03	**<.01**	10.40; 11.40
Female gender	0.84	0.02; 1.66	**.04**	2.28; 2.35	0.52	-0.12; 1.15	.11	1.58; 1.81	0.84	0.03; 1.66	**.04**	2.30; 2.39	0.49	-0.20; 1.18	.17	1.48; 1.76
BMI	-0.01	-0.07; 0.04	.67	0.98; 1.00	-0.03	-0.09; 0.04	.39	0.96; 1.00	0.003	-0.05; 0.06	.90	0.99; 1.02	-0.01	-0.08; 0.06	.76	0.97; 1.01
Age 43 to 51 years	0.16	-0.58; 0.89	.674	1.08; 1.23	0.29	-0.63; 1.21	.54	1.19; 1.43	0.13	-0.61; 0.86	.74	1.08; 1.18	0.26	-0.68; 1.19	.59	1.17; 1.39
52 to 57 years	-0.03	-0.80; 0.75	.95	0.90; 1.07	0.57	-0.39; 1.53	.25	1.60; 1.86	-0.12	-0.92; 0.69	.78	0.79; 0.99	0.44	-0.58; 1.46	.39	1.34; 1.71
58 years and older	-0.54	-1.22; 0.14	.12	0.56; 0.63	-.0.04	-0.82; 0.74	.92	0.87; 1.12	-0.61	-1.32; 0.10	.09	0.51; 0.59	-0.10	-0.91; 0.71	.80	0.79; 1.06

Predictors were Lean maturity in total at T1 (models 2a and 2b) or each of 4P's (model 2c and 2d); complaints at T2 were the outcome in all models. Gender, BMI and age were included as covariates in all models. Reference groups in the control variables were male and age ≤ 42; β, unstandardized beta coefficient; T1, time 1; T2 time, 2; 4P, Philosophy, Processes, People and partners, and Problem-solving; CI, confidence interval; P, p-value; OR, odds ratio; MI, multiple imputation.

For peer review only - http://bmjopen.bmj.com/site/about/guidelines.xhtml

Table 8. Association between Lean maturity and musculoskeletal complaints in the low back over time

**Low back complaints at T2**	**Model 2a, only Lean maturity in total and covariates**				**Model 2b, complaints T1 added**				**Model 2c, 4P (instead of Lean maturity in total)**				**Model 2d, Complaints T1 added**			
	β	95% CI	*P*	OR range; MI data set 1-5	β	95% CI	*P*	OR range; MI data set 1-5	β	95% CI	*P*	OR range; MI data set 1-5	β	95% CI	*P*	OR range; MI data set 1-5
Intercept				-1.34				-1.89				-1.60				-2.20
Lean maturity in total at T1	0.004	-0.02; 0.03	.74	1.00; 1.01	0.02	-0.01; 0.04	.26	1.01; 1.02								
Low back complaints at T1					2.00	1.44; 2.55	**<.01**	6.46; 8.64								
Philosophy									0.11	-0.03; 0.25	.13	1.09; 1.17	0.10	-0.07; 0.27	.25	1.07; 1.18
Processes									-0.05	-0.14; 0.04	.25	0.93; 0.97	-0.07	-0.19; 0.05	.24	0.90; 0.97
People and partners									0.06	-0.15; 0.26	.59	1.02; 1.16	0.15	-0.09; 0.39	.21	1.11; 1.28
Problem-solving									-0.02	-0.17; 0.13	.77	0.95; 1.00	0.001	-0.17; 0.17	.99	0.97; 1.03
Complaints at T1													2.07	1.50; 2.64	**<.01**	6.77; 9.43
Female gender	0.53	-0.32; 1.37	.22	1.51; 1.92	0.50	-0.51; 1.50	.33	1.37; 2.07	0.48	-0.37; 1.33	.27	1.46; 1.81	0.46	-0.57; 1.48	.38	1.28; 2.00
BMI	0.02	-0.05; 0.10	.53	1.01; 1.04	-0.01	-0.09; 0.07	.74	0.97; 1.00	0.04	-0.04; 0.11	.31	1.03; 1.05	0.004	-0.07; 0.08	.91	0.99; 1.02
Age 43 to 51 years	0.38	-0.28; 1.03	.26	1.36; 1.54	0.26	-0.40; 0.92	.44	1.25; 1.36	0.37	-0.32; 1.05	.29	1.36 1.52	0.24	-0.47; 0.94	.51	1.23; 1.32
52 to 57 years	0.18	-0.61; 0.96	.66	1.15; 1.25	0.38	-0.41; 1.17	.35	1.35; 1.76	0.13	-0.71; 0.97	.76	1.08; 1.25	0.28	-0.61; 1.18	.53	1.16 1.67
58 years and older	-0.13	-0.72; 0.46	.66	0.81; 0.96	-0.04	-0.68; 0.60	.91	0.88; 1.09	-0.17	-0.79; 0.44	.58	0. 78; 0.94	-0.08	-0.79; 0.63	.82	0.80; 1.04

Predictors were Lean maturity in total at T1 (models 2a and 2b) or each of 4P's (model 2c and 2d); complaints at T2 were the outcome in all models. Gender, BMI and age were included as covariates in all models. Reference groups in the control variables were male and age ≤ 42; β, unstandardized beta coefficient; T1, time 1; T2 time, 2; 4P, Philosophy, Processes, People and partners, and Problem-solving; CI, confidence interval; P, p-value; OR, odds ratio; MI, multiple imputation.

For peer review only - http://bmjopen.bmj.com/site/about/guidelines.xhtml

Table 9. Association between Lean maturity and musculoskeletal complaints in the upper back over time

**Upper back complaints at T2**	**Model 2a, only Lean maturity in total and covariates**				**Model 2b, complaints T1 added**				**Model 2c, 4P (instead of Lean maturity in total)**				**Model 2d, Complaints T1 added**			
	β	95% CI	*P*	OR range; MI data set 1-5	β	95% CI	*P*	OR range; MI data set 1-5	β	95% CI	*P*	OR range; MI data set 1-5	β	95% CI	*P*	OR range; MI data set 1-5
Intercept				-0.74				-0.91				-0.82				-0.94
Lean maturity in total at T1	0.002	-0.02; 0.02	.88	1.00; 1.01	0.003	-0.02; 0.03	.84	1.00; 1.01								
Upper back complaints at T1					2.17	1.43; 2.91	**<.01**	7.44; 9.50								
Philosophy									0.01	-0.14; 0.16	.86	0.95; 1.08	0.008	-0.16; 0.18	.93	0.96; 1.08
Processes									-0.04	-0.14; 0.06	.40	0.94; 0.98	-0.07	-0.20; 0.07	.32	0.90; 0.98
People and partners									0.13	-0.10; 0.36	.25	1.09; 1.24	0.15	-0.12; 0.42	.26	1.07; 1.32
Problem-solving									-0.03	-0.21; 0.15	.78	0.95; 0.99	0.01	-0.20; 0.23	.89	0.98; 1.06
Complaints at T1													2.21	1.42; 3.01	**<.01**	7.57; 10.56
Female gender	-0.004	-0.75; 0.74	.99	0.82; 1.15	-0.41	-1.39; 0.57	.41	0.53; 0.78	-0.05	-0.79; 0.70	.90	0.78; 1.11	-0.44	-1.42; 0.55	.38	0.52; 0.77
BMI	0.002	-0.07; 0.07	.96	0.99; 1.03	-0.02	-0.10; 0.066	.71	0.96; 1.01	0.01	-0.07; 0.09	.79	0.99; 1.04	-0.01	-0.10; 0.09	.91	0.97; 1.03
Age 43 to 51 years	0.14	-0.59; 0.87	.70	0.96; 1.39	0.32	-0.55; 1.19	.46	1.04; 1.84	0.15	-0.62; 0.92	.69	0.97; 1.42	0.34	-0.58; 1.26	.46	1.05; 1.85
52 to 57 years	-0.27	-1.13; 0.59	.54	0.72; 0.86	-0.29	-1.38; 0.81	.61	0.65; 0.85	-0.31	-1.19; 0.57	.49	0.67; 0.84	-0.36	-1.53; 0.81	.55	0.59; 0.85
58 years and older	-0.65	-1.35; 0.05	.07	0.47; 0.59	-0.57	-1.38; 0.24	.17	0.49; 0.68	-0.67	-1.39; 0.05	.07	0.46; 0.58	-0.60	-1.47; 0.27	.18	0.46; 0.68

For peer review only - http://bmjopen.bmj.com/site/about/guidelines.xhtml

Predictors were Lean maturity in total at T1 (models 2a and 2b) or each of 4P's (model 2c and 2d); complaints at T2 were the outcome in all models. Gender, BMI and age were included as covariates in all models. Reference groups in the control variables were male and age ≤ 42; β, unstandardized beta coefficient; T1, time 1; T2 time, 2; 4P, Philosophy, Processes, People and partners, and Problem-solving; CI, confidence interval; P, p-value; OR, odds ratio; MI, multiple imputation.

For peer review only

DISCUSSION

The 12-month prevalence of low back pain in hospital settings has been reported to be higher than what we found in the present study(22, 23, 36, 37), whereas the prevalence of shoulder complaints appears lower in hospitals(22,23). In a study of different occupations in Iran, the prevalence of low back pain among healthcare staff was 58%(37), i.e. larger than in our sample. Only one study was conducted in a Swedish setting (36). A study by Ribeiro et al.(26) from primary care in Portugal reported a 63% prevalence of low back complaints, i.e. considerably more than in our sample. Of particular note, we found that 6% to 9% of the participants had daily musculoskeletal complaints, and that 3% to 7% had been forced to change work tasks or jobs due to their complaints. A history of pain is strongly associated with current pain(38,39), and studies – however, mainly among blue-collar workers – have suggested uninterrupted, severe neck and shoulder pain over a long period of time to be associated with decreased workability and increased sick leave(40).

We did not find any general association between Lean maturity and musculoskeletal complaints. However, cross-sectional analyses of each of the 4P showed that Philosophy was positively associated with neck complaints, while People and partners had a negative association. In the longitudinal analysis, none of the 4P were associated with complaints in any of the four investigated body regions, except for People and partners, which was positively associated with neck complaints when the other 4P were controlled for and complaints at T1 were included as a covariate. These results are to some extent difficult to interpret, but they can be explained by earlier findings suggesting that both physical and psychosocial working conditions can be influenced by Lean maturity, and are risk factors for musculoskeletal complaints(17,31). Since very few studies have been conducted in primary care, we cannot compare our results to previous reports from that sector. A comparison of

For peer review only

our results with studies conducted in a hospital setting is less relevant, due to major differences in physical and psychosocial working conditions between working in a hospital(20,23–29) and in primary care(31).

Also, our results differ from studies of other occupational sectors than healthcare, where increased Lean has been identified as a risk factor for musculoskeletal complaints(17-19). Likely, the effect of Lean depends on the occupational sector, inasmuch as both working conditions and implementations of Lean will differ according to the occupational context. Thus, our results are difficult to compare with previous studies because they may have used other descriptions of Lean, and have investigated other ways of implementing Lean than those pertinent to primary care(1,4). We measured Lean maturity using the LiHcQ, which addresses all of Liker's 14 Lean principles(5), while most of the studies included in Mazzocato et al.'s(10) review of Lean in healthcare assessed Lean to a less comprehensive level. Another important aspect in the context of previous papers is the risk of publication bias, i.e. that published studies mainly report significant results, while leaving out non-significant results(41). Our results indicate that musculoskeletal complaints among primary care staff are a concern, especially as some staff members have daily complaints and some have to change jobs or work tasks due to these complaints.

We did not find any clear associations between Lean maturity and musculoskeletal complaints, but we encourage future research to assess both Lean maturity and musculoskeletal complaints over a more extended period of time than one year. Further research will allow for a deeper understanding of whether changes in Lean maturity and musculoskeletal complaints are associated, and if so, what elements of Lean that are particularly important risk factors.

For peer review only

Strengths and limitations

One limitation of the study is the use of a convenience sample, and the relatively low response rate(39); this limits the generalizability of our results. Using staff reports of Lean maturity and musculoskeletal complaints can also be viewed as a limitation, as responses may suffer from common-method bias related to the participants' attitudes toward their workplace, their general health, their tendency to give socially desirable answers, and inaccurate recall. On the other hand, staff members can be considered to have the best insights into their own health and working conditions. Our sample comprised mainly female registered nurses. However, this reflected well the composition of primary care staff in Sweden, where the most common profession is registered nurses(42), and even healthcare in general, where 88% of the registered nurses are women(43). Data concerning activities conducted by the primary care units to adopt Lean between T1 and T2 were not collected, which limits the interpretation of our results. A strength of the study was its longitudinal design, and the use of valid and, in general, consistent instruments for measuring Lean maturity(33) as well as complaints. An additional strength was the MI approach for handling missing data, since this procedure does not lead to loss of data, such as several other alternatives. However, a major limitation of our study is the follow-up time frame of only one year. It may be too short to identify changes in both musculoskeletal health and Lean maturity (figure 2).

CONCLUSIONS

The prevalence of musculoskeletal complaints among primary care staff was high and did not change during one year. The extent of Lean maturity was not associated with complaints, neither in cross-sectional analyses nor in a one-year predictive analysis.

For peer review only

Acknowledgements

We would like to thank all participants in the study, and their primary health care institutions.

Funding

This research was financially supported by the University of Gävle (no grant number). The funder had no role in the study design, data collection, analysis, data interpretation or the decision to submit the paper for publication.

Competing interests

None declared.

Contributors

The study was designed by MK, SEM, LB, HH and ME. Data were collected by MK and analyzed by MK, HH and ME. MK was primarily responsible for drafting the manuscript, which was critically revised by SEM, LB, HH and ME. All authors, i.e. MK, SEM, LB, HH and ME, read and approved the final manuscript.

Patient consent Not applicable.

Ethical approval

The study was approved by the Regional Ethical Review Board in Uppsala (Reg. no. 2014/525 and 2014/525/1).

Provenance and peer review

Not commissioned; externally peer reviewed.

Data availability statement

The data presented in this study are available from the corresponding author upon reasonable request. Individual data are not publicly available due to general data protection regulations (GDPR), and personal data ordinance (PDPO).

Open access

This is an open access article distributed in accordance with the Creative Commons Attribution Non Commercial (CC BY-NC 4.0) license, which permits others to distribute, remix, adapt, build upon this work non-commercially, and license their derivative works on different terms, provided the original work is properly cited, appropriate credit is given, any changes made indicated, and the use is non-commercial.

See:http://creativecommons.org/licenses/by-nc/4.0/.

For peer review only

References

1. D'Andreamatteo A, Ianni L, Lega F, Sargiacomo M. Lean in healthcare: A comprehensive review. *Health Policy*. 2014;119(9).

2. Holden RJ. Lean Thinking in emergency departments: a critical review. *Ann Emerg Med*. 2011;57(3):265–78.

3. Joosten T, Bongers I, Janssen R. Application of lean thinking to health care: issues and observations. *Int J Qual Health Care.* 2009;21(5):341–7.

4. Souza LB De. Trends and approaches in lean healthcare. *Leadersh Heal Serv.* 2009;22(2):121–39.

5. Liker JK. The Toyota Way. New York: McGraw-Hill; 2004.

6. Pettersen J. Defining lean production: some conceptual and practical issues. *TQM J.* 2009;21(2):127–42.

7. Langstrand J, Drotz E. The rhetoric and reality of Lean: a multiple case study. *Total Qual Manag Bus Excell.* 2015;:1–15.

8. Kaltenbrunner M, Mathiassen SE, Bengtsson L, et al. Lean maturity and quality in primary care. *J Health Organ Manag.* 2019;33(2):141–154

9. Axelsson, R. (2000). "The organizational pendulum: Healthcare management in Sweden 1865-1998." Scandinavian Journal of Public Health **28**(1): 47-53.

10. Mazzocato P, Savage C, Brommels M, et al. Lean thinking in healthcare: a realist review of the literature. *Qual Saf Heal Care.* 2010;25;19(5):376–382.

11. Mazzocato, P., Holden, R. J., Brommels, M., Aronsson, H., Bäckman, U., Elg, M., & Thor, J. (2012). How does lean work in emergency care? A case study of a lean-inspired intervention at the Astrid Lindgren Children's hospital, Stockholm, Sweden. BMC health services research, 12(1), 1-13.

12. Dellve L, Williamsson A, Strömgren M, et al. Lean implementation at different levels in Swedish hospitals: the importance for working conditions and stress. *Int J Hum Factors Ergon.* 2015;3(3–4):235–53.

13. Simons P, Backes H, Bergs J, et al. The effects of a lean transition on process times, patients and employees. *Int J Health Care Qual Assur.* 2017;30(2):103–18.

14. Jorma T, Tiirinki H, Bloigu R, et al. LEAN thinking in Finnish healthcare. *Leadersh Health Serv.* 2016;29(1).

15. Rees GH, Gauld R. Can lean contribute to work intensification in healthcare? *J Health Organ Manag.* 2017;31(3):369–84.

For peer review only

16. Arlbjørn JS, Freytag PV. Evidence of lean: a review of international peer-reviewed journal articles. *Eur Bus Rev.* 2013;25(2):174–205.

17. Koukoulaki T. The impact of lean production on musculoskeletal and psychosocial risks: An examination of sociotechnical trends over 20 years. *Appl Ergon.* 2014;45(2):198–212.

18. Landsbergis PA, Cahill J, Schnall P. The impact of lean production and related new systems of work organization on worker health. *J Occup Health Psychol.* 1999;4(2):108.

19. Brännmark M, Håkansson M. Lean production and work-related musculoskeletal disorders: overviews of international and Swedish studies. *Work.* 2012;41 Suppl 1:2321–8.

20. Sirisawasd S, Taptagaporn S, Boonshuyar C, et al. Interventions commonly used to prevent work-related musculoskeletal disorders among healthcare workers. *J Heal Res.* 2018;32(5):371–83.

21. Saberipour B, Ghanbari S, Zarea K, et al. Investigating prevalence of musculoskeletal disorders among Iranian nurses: A systematic review and meta-analysis. *Clin Epidemiol Glob Heal.* 2018;(7):513–8

22. Long MH, Johnston V, Bogossian F. Work-related upper quadrant musculoskeletal disorders in midwives, nurses and physicians: A systematic review of risk factors and functional consequences. *Appl Ergon.* 2012;43(3):455–67.

23. Freimann T, Pääsuke M, Merisalu E. Work-related psychosocial factors and mental health problems associated with musculoskeletal pain in nurses: a cross-sectional study. *Pain Res Manag.* 2016.

24. Kuorinka I, Jonsson B, Kilbom A, et al. Standardised Nordic questionnaires for the analysis of musculoskeletal symptoms. *Appl Ergon.* 1987;18(3):233–7.

25. Statens offentliga utredningar. Kommunaliserad hemsjukvård, SOU 2011:55 Available from https://www.regeringen.se/49b6a1/contentassets/1bb6a8565d0c40959035a1fa8106ef7b/ko mmunaliserad-hemsjukvard-sou-201155, accessed 09 April 2019.

26. Ribeiro T, Serranheira F, Loureiro H. Work related musculoskeletal disorders in primary health care nurses. *Appl Nurs Res.* 2017;33:72–7.

27. Courvoisierl DS, Genevayl S, Cedraschil C, et al. Job strain, work characteristics and back pain: a study in a university hospital. *Eur J Pain.* 2011;15(6):634–40.

28. Bongers PM, Ijmker S, Van den Heuvel S, et al. Epidemiology of work related neck and upper limb problems: psychosocial and personal risk factors (part I) and effective interventions from a bio behavioural perspective (part II). *J Occup Rehabil.* 2006;16(3):272–95.

29. Feng B, Liang Q, Wang Y, et al. Prevalence of work-related musculoskeletal symptoms of the neck and upper extremity among dentists in China. *BMJ Open.* 2014;4(12):e006451.

30. da Costa BR, Vieira ER. Risk factors for work-related musculoskeletal disorders: a systematic review of recent longitudinal studies. *Am J Ind Med.* 2010;53(3):285–323.

31. Jatobá A, Bellas HC, Bonfatti R, et al. Designing for patient risk assessment in primary health care: a case study for ergonomic work analysis. *Cogn Technol Work.* 2016;18(1):215–31.

For peer review only

32. Hauke, A., Flintrop, J., Brun, E., & Rugulies, R. (2011). The impact of work-related psychosocial stressors on the onset of musculoskeletal disorders in specific body regions: A review and meta-analysis of 54 longitudinal studies. Work & Stress, 25(3), 243-256.

33. Kaltenbrunner M, Bengtsson L, Mathiassen SE, et al. A questionnaire measuring staff perceptions of Lean adoption in healthcare: development and psychometric testing. *BMC Health Serv Res.* 2017;17(1):235.

34. Kaltenbrunner M, Bengtsson L, Mathiassen SE, et al. Staff perception of Lean, care-giving, thriving and exhaustion: a longitudinal study in primary care. *BMC Health Serv Res.* 2019;19(1):1–10.

35. Newman DA. Missing Data: Five Practical Guidelines. *Organ Res Methods.* 2014;17(4):372–411.

36. Wahlstedt K, Norbäck D, Wieslander G, et al. Psychosocial and ergonomic factors, and their relation to musculoskeletal complaints in the Swedish workforce. *Int J Occup Saf Ergon.* 2010;16(3):311–21.

37. Mehrdad R, Shams-Hosseini NS, Aghdaei S, et al. Prevalence of low back pain in health care workers and comparison with other occupational categories in Iran: A systematic review. *Iran J Med Sci.* 2016;41(6):467.

38. Panken G, Hoekstra T, Verhagen A, et al. Predicting chronic low-back pain based on pain trajectories in patients in an occupational setting: an exploratory analysis. *Scand J Work Environ Health.* 2016;42(6):520–7.

39. Smedley J, Inskip H, Trevelyan F, et al. Risk factors for incident neck and shoulder pain in hospital nurses. *Occup Environ Med.* 2003;60(11):864–9.

40. Hallman DM, Holtermann A, Dencker-Larsen S, et al. Are trajectories of neck–shoulder pain associated with sick leave and work ability in workers? A 1year prospective study. *BMJ Open.* 2019;9(3):e022006.

41. Baruch Y, Holtom BC. Survey response rate levels and trends in organizational research. *Hum relations.* 2008;61(8):1139–60.

42. Swedish Association of Local Authorities and Regions (SALAR). Professions in primary care. https://skl.se/ekonomijuridikstatistik/statistik/personalstatistik/personalenisiffror/tabellerla n dstingsanstalldpersonal2018.27838.html, accessed 08 September 2017.

43. The National Board of Health and Welfare. Statistics on Licensed Health Care Personnel 2016 and Workforce Status 2015. http://www.socialstyrelsen.se/publikationer2018/2018-210,accessed 08 September 2017.

Figure 1. Flowchart showing staff members eligible for the present study, and the flow of responding staff members between T1 and T2.
T1, baseline (2015); T2, follow-up (2016).

For peer review only

Figure 2. Changes in individual ratings of Lean maturity between T1 and T2 for non-imputed data (n=104)

The value 0 indicates no change in rating between T1 and T2. Negative and positive values show a decrease and an increase, respectively, in Lean maturity from T1 to T2.

For peer review only

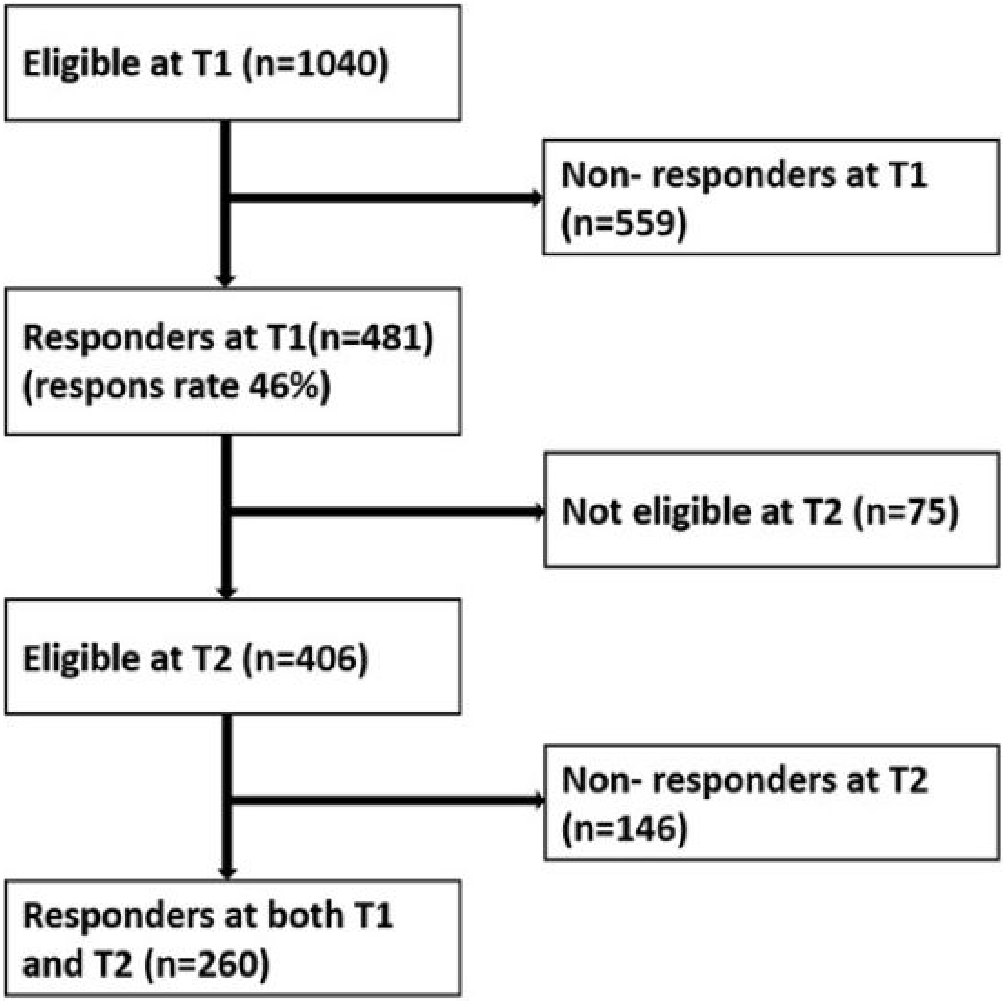

Figure 1 Flowchart showing staff members eligible for the present study, and the flow of responding staff members between T1 and T2. T1, baseline (2015); T2, follow-up (2016).

46x46mm (300 x 300 DPI)

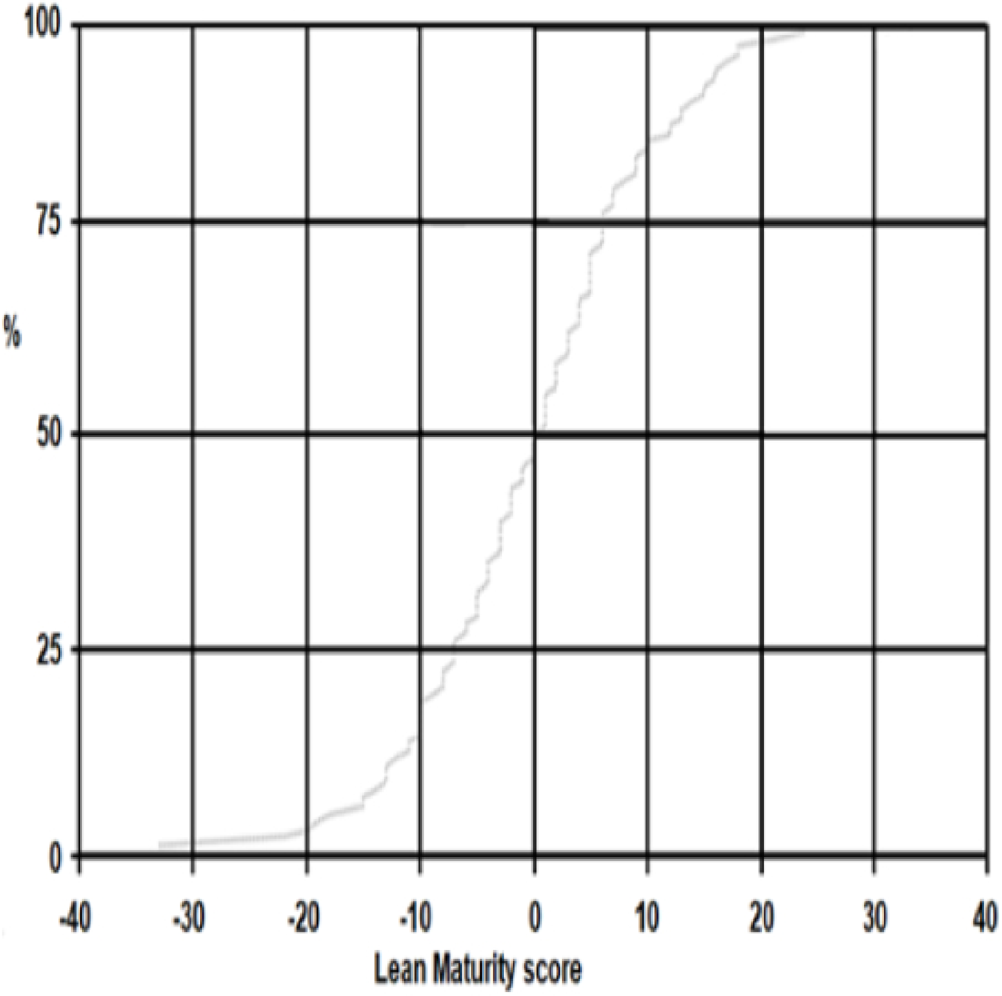

Figure 2 Changes in individual ratings of Lean maturity between T1 and T2 for non-imputed data (n=104). The value 0 indicates no change in rating between T1 and T2. Negative and positive values show a decrease and an increase, respectively, in Lean maturity from T1 to T2.

118x118mm (118 x 118 DPI)

STROBE Statement—Checklist

Referring to the draft received from BMJ open.

Titel: Associations between Lean Maturity in primary care and musculoskeletal complaints among staff. A longitudinal study.

	Item No	Recommendation	Page No	Row
**Title and abstract**	1	(*a*) Indicate the study's design with a commonly used term in the title or the abstract	2	5
		(*b*) Provide in the abstract an informative and balanced summary of what was done and what was found	3,<br>4	5-49<br>3-6
**Introduction**				
Background/rationale	2	Explain the scientific background and rationale for the investigation being reported	5,<br>6,<br>7,<br>8	6-50<br>3-50<br>3-50<br>3-15
Objectives	3	State specific objectives, including any prespecified hypotheses	8 (aim)	8-15
**Methods**				
Study design	4	Present key elements of study design early in the paper	8	24
Setting	5	Describe the setting, locations, and relevant dates, including periods of recruitment, exposure, follow-up, and data collection	8,<br>9	24-50<br>3-33
Participants	6	(*a*) Give the eligibility criteria, and the sources and methods of selection of participants	8,<br>9	45-50<br>3-8, fig 1
Variables	7	Clearly define all outcomes, exposures, predictors, potential confounders, and effect modifiers. Give diagnostic criteria, if applicable	9,<br>10	40-50<br>3-41
Data sources/ measurement	8*	For each variable of interest, give sources of data and details of methods of assessment (measurement). Describe comparability of assessment methods if there is more than one group	9,<br>10	40-50<br>3-41
Bias	9	Describe any efforts to address potential sources of bias	19-20	15-38
Study size	10	Explain how the study size was arrived at		
Quantitative variables	11	Explain how quantitative variables were handled in the analyses. If applicable, describe which groupings were chosen and why	10,<br>11,	47-50<br>3-29, Table1
Statistical methods	12	(*a*) Describe all statistical methods, including those used to control for confounding	11,<br>12	3-29, Table1<br>6-21
		(*b*) Describe any methods used to examine subgroups and interactions	10,<br>11,	50<br>3-29 Table 1
		(*c*) Explain how missing data were addressed	12	6-21
		(*d*) If applicable, describe analytical methods taking account of sampling strategy		
		(*e*) Describe any sensitivity analyses		

Results

For peer review only - http://bmjopen.bmj.com/site/about/guidelines.xhtml

Participants	13*	(a) Report numbers of individuals at each stage of study—eg numbers potentially eligible, examined for eligibility, confirmed eligible, included in the study, completing follow-up, and analysed	34	Figure 1
		(b) Give reasons for non-participation at each stage	34	
		(c) Consider use of a flow diagram	34	
Descriptive data	14*	(a) Give characteristics of study participants (eg demographic, clinical, social) and information on exposures and potential confounders	13	Table 2
		(b) Indicate number of participants with missing data for each variable of interest		
Outcome data	15*	Report numbers of outcome events or summary measures	11	15-20
Main results	16	(*a*) Give unadjusted estimates and, if applicable, confounder-adjusted estimates and their precision (eg, 95% confidence interval). Make clear which confounders were adjusted for and why they were included	16-17	Table 5-9
		(*b*) Report category boundaries when continuous variables were categorized		
		(*c*) If relevant, consider translating estimates of relative risk into absolute risk for a meaningful time period		
Other analyses	17	Report other analyses done—eg analyses of subgroups and interactions, and sensitivity analyses	11	3-27
**Discussion**				
Key results	18	Summarise key results with reference to study objectives	17, 18, 19	29-51 3-50 3-38
Limitations	19	Discuss limitations of the study, taking into account sources of potential bias or imprecision. Discuss both direction and magnitude of any potential bias	19, 20	44-51 3-24
Interpretation	20	Give a cautious overall interpretation of results considering objectives, limitations, multiplicity of analyses, results from similar studies, and other relevant evidence	17, 18, 19, 20	30-51 3-50 3-38 3-24
Generalisability	21	Discuss the generalisability (external validity) of the study results	18, 19	38-60 1-15, 44-46
**Other information**				
Funding	22	Give the source of funding and the role of the funders for the present study and, if applicable, for the original study on which the present article is based	21	7-8

For peer review only - http://bmjopen.bmj.com/site/about/guidelines.xhtml
